# Multidimensional hyperspin machine

**DOI:** 10.1038/s41467-022-34847-9

**Published:** 2022-11-25

**Authors:** Marcello Calvanese Strinati, Claudio Conti

**Affiliations:** 1 Centro Ricerche Enrico Fermi (CREF), Via Panisperna 89a, 00184 Rome, Italy; 2 Institute for Complex Systems, National Research Council (ISC-CNR), 00185 Rome, Italy; 3 Physics Department, Sapienza University of Rome, 00185 Rome, Italy

**Keywords:** Nonlinear optics, Information theory and computation

## Abstract

From condensed matter to quantum chromodynamics, multidimensional spins are a fundamental paradigm, with a pivotal role in combinatorial optimization and machine learning. Machines formed by coupled parametric oscillators can simulate spin models, but only for Ising or low-dimensional spins. Currently, machines implementing arbitrary dimensions remain a challenge. Here, we introduce and validate a hyperspin machine to simulate multidimensional continuous spin models. We realize high-dimensional spins by pumping groups of parametric oscillators, and show that the hyperspin machine finds to a very good approximation the ground state of complex graphs. The hyperspin machine can interpolate between different dimensions by tuning the coupling topology, a strategy that we call “dimensional annealing”. When interpolating between the XY and the Ising model, the dimensional annealing substantially increases the success probability compared to conventional Ising simulators. Hyperspin machines are a new computational model for combinatorial optimization. They can be realized by off-the-shelf hardware for ultrafast, large-scale applications in classical and quantum computing, condensed-matter physics, and fundamental studies.

## Abstract (web summary)

Spin simulators can solve many combinatorial optimization problems that can be represented by spin models, but they are limited to low-dimensional spins. Here the authors propose a simulator of multidimensional spins in arbitrary dimension, using a system of coupled parametric oscillators with a common pump.

## Introduction

Systems of interacting spins are ubiquitous in nature. Their complex collective behavior and their equilibrium properties describe magnetism in solid-state systems^1^, phase transitions in spin glasses^2^, quantum chromodynamics (QCD)^3^, and quantum and classical computation^4^. Spin models are also pivotal in combinatorial optimization^5^, with applications in machine learning^6^, traffic and portfolio optimization^7^, markets and finance^8^, biology and life science^9^, artificial intelligence^10^, protein folding^11^, epidemic spreading^12^, bioinformatic^13^, and material engineering^14^.

However, simulating and understanding spin systems remain a challenge, as several models are computationally (NP-)hard^15^. Novel algorithms and techniques are emerging, including the realization of specialized physical machines that converge to the ground state (GS) of programmable spin Hamiltonians, a major quest in the last decades^16–42^. However, most of the work has been limited to the simulation of one-component, discrete spin systems (Ising model), and of continuous spin models with two or three components (XY or Heisenberg models, respectively). Also, spin machines, either software or hardware, suffer of limitations as heterogeneity and stiffness, which reduce the success probability in computationally NP-hard models to a narrow range of parameters. Heterogeneity refers to the fact that the spin simulator exhibits local energy minima not present in the target model. Stiffness appears in binary models that display deep local energetic states, which impede reaching the ground state during minimization or annealing. Ideally, one would use high-dimensional systems to increase the symmetry in order to connect the many local minima and interpolate binary spins with continuous variables to exit the energetic traps. However, these features are not available at the moment.

Ising spin simulators include two-component Bose-Einstein condensates^17,18^, superconducting circuits^19^, digital computers^20–22^, electrical oscillators^23^, optoelectronical oscillators^24^, and degenerate optical parametric oscillators (POs)^25–34^ forming a coherent Ising machine (CIM). Proposed platforms to simulate classical XY models include laser networks^35,36^, non-degenerate POs^37^, and polariton condensates^38^. Quantum spin simulators include trapped atomic ion crystals for the quantum Ising, XY, and Heisenberg models^39–42^.

Programmable multicomponent spins represent a toolbox for the systematic study of nontrivial phases, symmetry breaking phenomena, as well as the critical behavior of phase transitions in condensed-matter physics^3^. Examples include magnetic properties of three-dimensional spins^43^ and critical properties of spin glasses^44^. Importantly, classical multidimensional spins may simulate the behavior of quantum many-body systems^45^. Four-dimensional spin models appear in QCD, describing the symmetry and critical properties of the chiral phase transition with two light-quark flavors^3,46–48^.

In this article, we propose and validate a classical simulator of a *N*-spin system with an arbitrary number *D* of spin components. The rationale behind our proposal can be summarized as follows. The hyperspin machine is realized by coupling *D* × *N* POs in a hierarchical way: First, the network is organized as *N* POs multiplets by using *N* identical pump fields, each one driving a group of *D* POs. Within each group, the common pump saturation induces an effective nonlinear coupling between the POs. We will show that the steady state of the resulting dynamics allows to construct from each PO multiplet a *D*-dimensional continuous spin (or hyperspin), where a single PO represents a component of the hyperspin. On top of this structure, a linear coupling between POs in different multiplets realizes the hyperspin system. The linear coupling identifies the actual hyperspin-hyperspin coupling, and the resulting network of nonlinearly and linearly coupled POs simulates a system of *N*, *D*-dimensional, hyperspins. The choice of POs as fundamental constituents of an hyperspin is motivated by the fact that they furnish a versatile platform to realize artificial spin devices at room temperature. POs grant an extraordinary degree of control and the prospect to realize scalable systems of coupled all-optical POs with size-independent ultra-fast equilibration times^49^. However, despite their potential use as physical hardware, we show here that even only the software implementation of an hyperspin machine enables a novel strategy for annealing that we call “dimensional annealing”, which increases the probability to optimize hard models in a wide range of parameters.

## Results

### Multidimensional hyperspin with POs

**Fig. 1:**
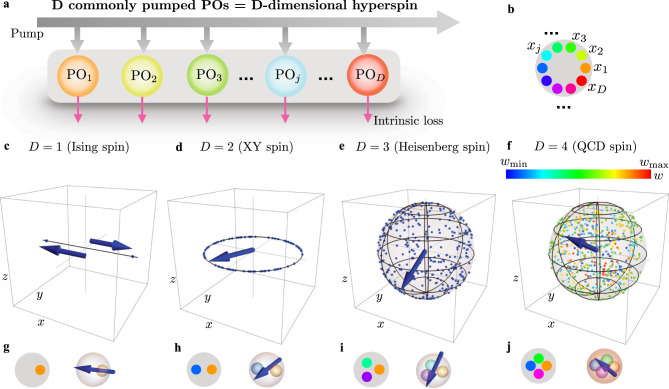
Composite parametric oscillator (PO) as a *D*-dimensional hyperspin. **a** The spin consists of *D* degenerate POs (colored dots) saturating the same pump field (gray arrows and area) with equal intrinsic loss (purple arrows). **b** Compact representation of the scheme in **a**. The colored dots denote the *D* POs, described by dynamical variables *x*_1_, …*x*_*D*_, and the gray circle represents the common pump. **c**, **d**, **e**, **f** Fixed points of the composite PO system. The fixed points lie on the surface of a *D*-dimensional hypersphere. For *D* = 1, there are two fixed points on the *x*-axis representing the two states of an Ising spin; For *D* = 2, the fixed points lie on a circumference in the *xy*-plane, encoding the continuous phase of a XY spin; For *D* = 3, the fixed points lie on the surface of a sphere in the *xyz*-space, encoding the two angles of an Heisenberg spin; For *D* = 4, the fixed points are represented by encoding three of the four coordinates into a point within the volume of a sphere in the *xyz*-space, and the fourth coordinate *w* is encoded as a color with extremal values *w*_m*in*_ and *w*_m*ax*_ in the colormap. **g**, **h**, **i**, **j** Left panels are the composite PO representation of the *D*-dimensional spin as in panel **b**, while right panels are a three-dimensional representation of the composite PO as a spin $\overrightarrow{\sigma }$ in standard hyperspherical coordinates (blue arrow). For *D* = 4, borrowing the terminology from QCD, the arrow represents the “vector” *xyz*-component of the QCD spin, and the color assigned to the outer sphere encodes the “scalar” *w*-component.

We start by showing how an hyperspin is constructed from *D* commonly pumped POs. We consider in Fig. 1a, b *D* identical POs, all with frequency *ω*_0_ and loss *g*, described by classical dynamical variables *x*_1_, …*x*_*D*_ pumped by an external drive with amplitude *h* and frequency 2*ω*_0_. The pump feeds the *D* oscillators, with saturation value *h*(1 − *βI*), where *β* is a saturation coefficient and $I={\sum }_{l}{x}_{l}^{2}$ is the total PO energy. Following the formalism in refs. 30, 50, 51, we describe the dynamics by *D* coupled Mathieu’s equations

1 $${\ddot{x}}_{j}\,+\,{\omega }_{0}^{2}\,\left[1\,+\,gh\,\left(1\,-\,\beta \mathop{\sum }\limits_{l=1}^{D}{x}_{l}^{2}\right)\sin (2{\omega }_{0}t)\right]\,{x}_{j}\,+\,{\omega }_{0}g{\dot{x}}_{j}\,=\,0,$$

where *j* = 1, …, *D* labels the different POs. Differently from refs. 30, 50, 51 and related literature, POs in Eq. (1) are not coupled by a conventional linear coupling. Instead, the common pump saturation induces an effective nonlinear coupling between POs. When pumped above the threshold value *h*_th_, each PO *x*_*j*_ responds with an oscillation at frequency locked to half the pump frequency due to period doubling instability. This oscillation is modulated by a complex amplitude *X*_*j*_, which describes the nontrivial dynamics of the PO variable *x*_*j*_. When an amplitude steady-state exists, the fixed points ${\overline{X}}_{j}=|{\overline{X}}_{j}|{e}^{i{\phi }_{j}}$ encode the equilibrium values of the magnitude and phases of the PO fast oscillations, ${\overline{x}}_{j}(t)=2|{\overline{X}}_{j}|\cos ({\omega }_{0}t+{\phi }_{j})$.

The dynamics of the complex amplitudes *X*_*j*_ is found from Eq. (1) by a multiple-scale expansion^50,52^ (see Methods). For a range of *h* values above threshold, the dynamics amplifies the amplitude real parts, and suppresses the imaginary parts. The fixed-point values ${\overline{X}}_{j}:=\mathop{\lim }\nolimits_{t\to \infty }{X}_{j}(t)$ are real numbers, i.e., the phase *ϕ*_*j*_ is binary (either 0 or *π*). In units such that *ω*_0_ = 1, the time evolution of the amplitudes reads

2 $$\frac{\partial {X}_{j}}{\partial t}=\left(\frac{h}{4}-\frac{1}{2}-\frac{h\beta }{2}\mathop{\sum }\limits_{l=1}^{D}{X}_{l}^{2}\right){X}_{j}.$$

The reason why the PO system in Eq. (2) can describe a *D*-dimensional spin follows from the fixed-point configuration of the amplitude dynamics, which are found as customary by equating Eq. (2) to zero. This implies $\mathop{\sum }\nolimits_{l=1}^{D}{\overline{X}}_{l}^{2}={S}^{2}$ where $S=\sqrt{\mathop{\sum }\nolimits_{l=1}^{D}{\overline{X}}_{l}^{2}}= \sqrt{(1/2-1/h)/\beta }$. Thus, ${\{{\overline{X}}_{j}\}}_{j=1}^{D}$ are the Cartesian coordinates of a point on a *D*-dimensional hypersphere, and the corresponding unit vector is a continuous, *D*-dimensional hyperspin, i.e., $\overrightarrow{\sigma }=({\overline{X}}_{1},\ldots,\,{\overline{X}}_{D})/S$.

To clarify the connection between the PO system in Eq. (1) and a continuous *D*-dimensional spin, we show in Fig. 1c, d, e, f the configuration of the fixed points for the specific cases *D* = 1, 2, 3, 4. We numerically integrate Eq. (2) using different random initial conditions. At the end of each integration, we obtain the real coordinates ${\{{\overline{X}}_{j}\}}_{j=1}^{D}$, and plot them in the *xyz*-space as blue or colored dots as follows: For *D* = 1 (panel c), one has a single PO with two fixed points that describe the two values of an Ising spin. In our notation, the PO fixed-point quadrature identifies the *x*-coordinate, and the *y*- and *z*- coordinates are set to zero. For *D* = 2 (panel d), the two quadratures take any value on a circumference, and they identify the *x*- and *y*- coordinates (the *z*-coordinate is set to zero). The corresponding unit vector defines an XY spin. For *D* = 3 (panel e), the three PO quadratures take any value on the surface of a sphere (the *z*-coordinate being identified by ${\overline{X}}_{3}$), and the unit vector defines an Heisenberg spin. For *D* = 4 (panel f), a fixed point has four coordinates on the surface of a four-dimensional hypersphere. We plot the three-dimensional projected vector $({\overline{X}}_{1},\,{\overline{X}}_{2},\,{\overline{X}}_{4})$ within the volume of a three-dimensional sphere of radius *S*, and the extra quadrature ${\overline{X}}_{3}$ defines the fourth coordinate *w* whose value is encoded as a color. Following the conventionally adopted terminology in QCD, we name the four-dimensional unit vector as a QCD spin, where the projected vector in the *xyz*-space is the “vector” and ${\overline{X}}_{3}$ is the “scalar” field^47,53–55^.

Left panels in Fig. 1g, h, i, j show the composite PO as in panel b, and right panels give a three-dimensional representation of the spin $\overrightarrow{\sigma }$ in standard hyperspherical coordinates with unit radius^56,57^: The sign of the PO quadrature for *D* = 1, and polar and spherical coordinates of the quadrature unit vector for *D* = 2 and *D* = 3 respectively. For *D* = 4, the arrow represents the “vector” component in three-dimensional spherical coordinates, while the additional angle encoding the “scalar” coordinate is a color assigned to the outer sphere.

### Coupled *D*-dimensional hyperspins

We now move to the case of coupled composite POs and explicit the relation between the network dynamics and the *D*-vector spin model Hamiltonian^58^

3 $${H}_{D}(\{\overrightarrow{\sigma }\})=-\mathop{\sum }\limits_{q,p=1}^{N}{J}_{qp}\,{\overrightarrow{\sigma }}_{q}\cdot {\overrightarrow{\sigma }}_{p},$$

with non-uniform hyperspin-hyperspin coupling quantified by the adjacency matrix **J**. Starting from Eq. (1) for the single *D*-dimensional hyperspin, we model the PO network simulating *N* coupled hyperspins by the *D* × *N* classical equations of motion

4 $${\ddot{x}}_{j}\,+\,\left[1\,+\,gh\,\left(\,1\,-\,\beta \mathop{\sum }\limits_{l=1}^{DN}{W}_{jl}{x}_{l}^{2}\,\right)\,\sin (2t)\right]\,{x}_{j}\,-\,g\mathop{\sum }\limits_{l=1}^{DN}{C}_{jl}{\dot{x}}_{l}\,=\,0.$$

**Fig. 2:**
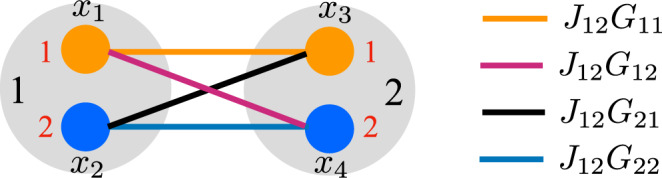
PO connectivity as hyperspins. We show here the connectivity for *N* = *D* = 2. The POs *x*_*j*_ with *j* = 1, 2, 3, 4 form two spins labeled by *q* = 1, 2 (black labels) with two components *μ* = 1, 2 each (red labels), where the indexes are related as $\mu=1+(j-1){{{{{{{\rm{mod}}}}}}}}(D)$ and *q* = 1 + ⌊(*j* − 1)/*D*⌋. The indexes *j* are then grouped as ${{\mathbb{S}}}_{1}=\{1,\,2\}$ and ${{\mathbb{S}}}_{2}=\{3,\,4\}$. The linear coupling term *C*_*jl*_ between *x*_*j*_ and *x*_*l*_ with *j* ≠ *l* is decomposed as *C*_13_ = *J*_12_*G*_11_, *C*_14_ = *J*_12_*G*_12_, *C*_23_ = *J*_12_*G*_21_, and *C*_24_ = *J*_12_*G*_22_ (see legend), while *C*_12_ = *C*_34_ = 0. The amplitudes ${X}_{1}\equiv {X}_{1}^{(1)}$ and ${X}_{2}\equiv {X}_{2}^{(1)}$, and ${X}_{3}\equiv {X}_{1}^{(2)}$ and ${X}_{4}\equiv {X}_{2}^{(2)}$ form the *μ* = 1, 2 components of the *q* = 1 and *q* = 2 hyperspins, respectively, ${\overrightarrow{S}}_{1}=({X}_{1},\,{X}_{2})$ and ${\overrightarrow{S}}_{2}=({X}_{3},\,{X}_{4})$.

In Eq. (4), we define two different couplings: The matrix **W** describes the nonlinear coupling that organizes the POs as *N* multiplets of *D* commonly pumped POs, and the matrix **C** defines the linear coupling stabilizing the hyperspin network. The off-diagonal element *C*_*jl*_ quantifies the coupling strength between POs *x*_*j*_ and *x*_*l*_, while *C*_*jj*_ = − 1 identifies the intrinsic loss for the *j*-th PO. In this arrangement, the *q*-th hyperspin is identified by the PO indexes $j\in {{\mathbb{S}}}_{q}$ with ${{\mathbb{S}}}_{q}:=\{1+(q-1)D,\ldots,\,qD\}$, where each PO amplitude *X*_*j*_ within this set identifies the *μ*-th component of the *q*-th hyperspin vector ${\overrightarrow{S}}_{q}$ as ${X}_{j}\to {X}_{\mu+(q-1)D}\equiv {X}_{\mu }^{(q)}$ (see Fig. 2 for a pictorial representation with *N* = *D* = 2). The coupling matrix can in general be written as the sum of a symmetric and antisymmetric part, identifying the dissipative and energy-preserving part of the coupling, respectively^50,51^. A dissipative coupling is commonly considered when using POs for optimiziation^59^, while the energy-preserving coupling proposed in^30^ inducing persistent coherent beats between POs has been recently used to realize photonic spiking neurons^60^. Hereafter, we focus on symmetric coupling matrices.

**Fig. 3:**
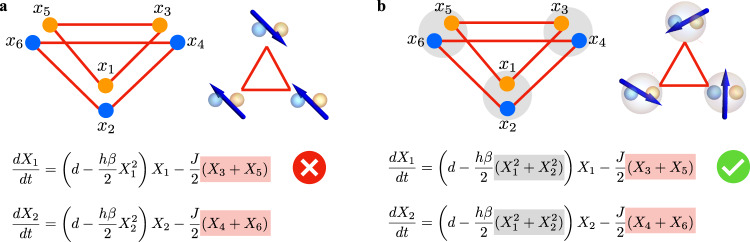
Hierarchical coupling to construct the hyperspin machine. Specifically, we show here an *N* = 3 XY machine (*D* = 2) with antiferromagnetic linear coupling (*J*_*qp*_ = − *J* and ${{{{{{{\bf{G}}}}}}}}={{\mathbb{1}}}_{2}$, red lines and areas). We report the equations of motion for *X*_1_ and *X*_2_ [*d* = *h*/4 − 1/2, see Eq. (5)] for completeness. **a** Without common pump saturation (i.e., the pump for the *X*_*j*_ PO is saturated by ${X}_{j}^{2}$ only), the system consists of two disjoint PO networks (there is no coupling between *X*_1,3,5_ and *X*_2,4,6_), where each network is an Ising simulator yielding *σ*_*j*_ = ± 1. In this way, no continuous spin is defined following Fig. 1. **b** In contrast to **a**, with common pump saturation (gray dots and area) POs within each multiplet are now effectively coupled by a nonlinear coupling. This nonlinear coupling makes each PO multiplet suitable to represent an hyperspin (see Fig. 1). In addition to this nonlinear coupling, the linear coupling between POs in different multiplets realizes the hyperspin machine.

The equations for the slowly varying amplitudes *X*_*j*_ from Eq. (4) are detailed in Supplementary Note 2. Figure 2 shows the arrangement as *N* hyperspins with *D* POs. We decompose the coupling matrix as **C** = **J** ⊗ **G**, where **J** is the *N* × *N* adjacency matrix encoding the specific multidimensional spin model, and **G** is a *D* × *D* metric tensor. With this choice of **C** and redefinition of the indexes, when the dynamics of the PO amplitudes suppresses their imaginary parts, one can write ($j\in {{\mathbb{S}}}_{q}$)

5 $$\frac{d{X}_{\mu }^{(q)}}{dt}\,=\,\left(\,\frac{h}{4}\,-\,\frac{1}{2}\,-\,\frac{h\beta }{2}\,{S}_{q}^{2}\right)\,\,{X}_{\mu }^{(q)}+\frac{1}{2}\mathop{\sum }\limits_{p=1}^{N}\mathop{\sum }\limits_{\nu=1}^{D}\,{J}_{qp}\,{G}_{\mu \nu }{X}_{\nu }^{(p)},$$

where ${S}_{q}^{2}={\sum }_{l\in {{\mathbb{S}}}_{q}}{X}_{l}^{2}$ denotes the squared amplitude of the *q*-th hyperspin. These amplitudes are unconstrained, and in the steady-state different hyperspins may have different *S*_*q*_ (amplitude heterogeneity)^61^. This issue causes the dynamical system to minimize a cost function (coupled oscillators) that in general differs from the desired one (coupled spins)^59,62–64^, eventually spoiling the quality of the solution. The key observation is that, for a given adjacency matrix **J** and in the proper regime of pump amplitude *h* above *h*_th_, amplitude heterogeneity is reduced and the PO network in Eq. (5) behaves as a gradient descendent system driving the spin configuration towards the minimum of the *D*-vector spin model Hamiltonian in Eq. (3) when ${{{{{{{\bf{G}}}}}}}}={{\mathbb{1}}}_{D}$ and with the spin vectors ${\overrightarrow{\sigma }}_{q}={\overrightarrow{S}}_{q}/{S}_{q}$ (see Methods and Supplementary Note 2 for the proof). Figure 3 exemplifies the construction of the hyperspin machine from the nonlinear and linear couplings [Eqs. (4) and (5) specifically to realize an XY machine (*D* = 2) with *N* = 3 antiferromagnetically coupled XY spins], highlighting the necessity of common pump saturation. For completeness, we also show the equations of motion for the POs in the first hyperspin (*X*_1_ and *X*_2_). In the absence of nonlinear coupling between POs *X*_*j*_ and *X*_*j*+1_ with *j* = 1, 3, 5 [panel (a)], the system consists of *D* disjoint Ising simulators (one identified by *X*_1_, *X*_3_, and *X*_5_, and the other one by *X*_2_, *X*_4_, and *X*_6_), each one simulating the Ising model encoded in the linear coupling. This case does not realize the hyperspin machine. Instead, when POs *X*_*j*_ and *X*_*j*+1_ (*j* = 1, 3, 5) saturate the same pump [panel (b)], they become nonlinearly coupled, forming an hyperspin [Eq. (2)], and the *D* × *N* = 6 POs are arranged as *N* = 3 XY spins. The linear coupling between POs in different hyperspins realizes the hyperspin network, and the resulting interplay between nonlinear and linear coupling induces a nontrivial dynamics that encodes the *D*-vector spin model, which in this case simulates three coupled XY spins.

**Fig. 4:**
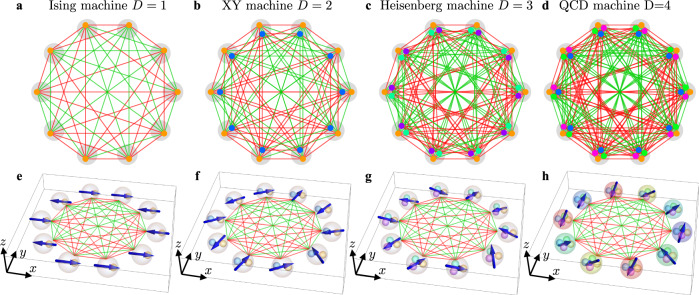
Network of *D* × *N* POs simulating *N*, *D*-dimensional hyperspins. The coupling represents here a random complete K graph. The network is shown with *N* = 10 and embedded in a circular geometry. **a**, **b**, **c**, **d** Full PO network for *D* = 1, 2, 3, 4 as in the legends. Green and red lines represent positive end negative entries of the adjacency matrix **J**, respectively. **e**, **f**, **g**, **h** Hyperspin representation in the *xyz*-space of the PO network, where hyperspins are represented as in Fig. 1. The steady-state values of the real-part of the PO amplitudes dynamics *X*_*j*_(*t*) from the numerical integration of Eq. (5) determine the state of the spins (see Supplementary Note 2 and Supplementary Movie 1).

**Fig. 5:**
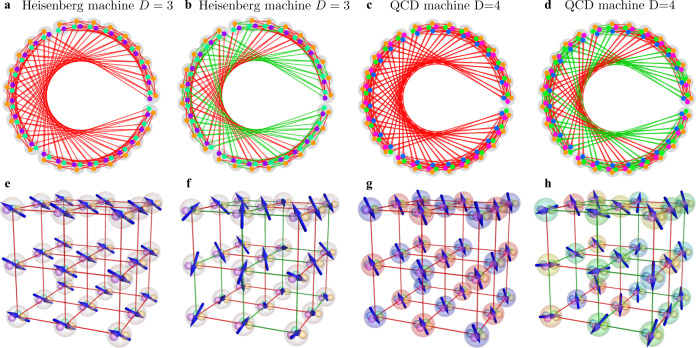
Hyperspins in solid topology. Network of *D* × *N* composite POs representing a three-dimensional solid topology with nearest-neighbor interaction (spin glass) of *N* = *N*_*x*_ × *N*_*y*_ × *N*_*z*_ = 27 spins, specifically with *N*_*x*_ = *N*_*y*_ = *N*_*z*_ = 3, and *D* = 3, 4 as in the legends. **a**, **b**, **c**, **d**, PO network connectivity in circular embedding for **a**, **c** uniform antiferromagnetic interaction, and **b**, **d** random binary interaction. **e**, **f**, **g**, **h**, Hyperspin network representation with solid embedding of the PO connectivity in panels **a**, **b**, **c**, **d**. The final hyperspin state is retrieved as in Fig. 4, see also Supplementary Movies 2 and 3.

We show in Figs. 4 and 5 two prototype examples of PO connectivity and equivalent representation as hyperspins in the *xyz*-space. In Fig. 4, we consider a random complete (K) graph^65^ with *N* = 10 spins for dimension *D* = 1, 2, 3, 4. The adjacency matrix has entries with fixed amplitude ∣*J*_*qp*_∣ = 0.03 and sign randomly chosen with equal probability for each *q* and *p*. The case *D* = 1 in panel a represents the Ising model, and Eq. (5) gives the PO dynamics of CIMs^66^. In this case, each spin takes a binary value, represented by an oriented arrow along the *x*-axis in panel e. The spin state is retrieved from the steady-state values of the PO amplitudes ${\overline{X}}_{j}\equiv {\overline{X}}_{\mu }^{(q)}$ from the numerical integration of the complex amplitude equations, whose real-part evolution is Eq. (5), seeded with a random complex initial condition *X*_*j*_(0) (see Supplementary Note 2 and Supplementary Movies 1-3). The higher-dimensional cases in panels b, c, d for *D* = 2, 3, 4 simulate the XY, Heisenberg, and QCD model, respectively. The PO connectivity ${{{{{{{\bf{C}}}}}}}}={{{{{{{\bf{J}}}}}}}}\otimes {{\mathbb{1}}}_{D}$ for the scalar product in Eq. (3) is obtained by connecting a dot of a given color within a multiplet (gray circle) to the dot of the same color in another multiplet. The spin states in panels f, g, h are the representation of the spin vectors in standard hyperspherical coordinates^56,57^ (see Fig. 1). In all these cases, the random orientation of the spins reflects the disordered nature of the graph.

In Fig. 5, we show a hyperspin glass^2^, i.e., a solid three-dimensional system of *N* spins in the *xyz*-space in dimension *D* = 3 and *D* = 4 with nearest-neighbor interaction, arranged as a lattice of *N* = *N*_*x*_ × *N*_*y*_ × *N*_*z*_ hyperspins. Panels a, e and c, g consider a uniform antiferromagnetic interaction, while panels b, f and d, h are with a random binary interaction, where as for the K graphs ∣*J*_*qp*_∣ is fixed and its sign is randomly chosen with equal probability. The spin state is obtained as in Fig. 4. For the antiferromagnetic interaction, we obtain from our simulations an antiferromagnetically oriented spin structure, represented by the arrows both for *D* = 3 and *D* = 4 with additional alternating sphere colors. For the other cases (panels b, f and d, h), as for the K graph in Fig. 4, the spin orientation is random due to the disordered interaction. It is important to remark that general spin models have impact in many fields. Notable examples include the Ising^67^ and the Heisenberg spin glass^44^ for *D* = 1 and *D* = 3, respectively, and the finite-temperature phase transition in QCD with two light-quark flavors for *D* = 4^3,46–48^.

### Hyperspin Hamiltonian minimization

We now explicit the working principle of the hyperspin network simulator. We study specifically the *D*-vector model in Eq. (3). The PO network dynamics in Eq. (5) for general *D* drives the system close to the ground state of the *D*-vector Hamiltonian, sharing similarities with the conventional Ising simulators for *D* = 1, but with important differences. The hyperspin structure of *D* multiplet POs is given by the nonlinear coupling due to common pump saturation. For a pump amplitude *h* slightly above the threshold, nonlinearities affect the dynamics on a time scale much slower than the rate of energy exchange due to the linear coupling^27,63^. The PO amplitudes *X*_*j*_ freeze to the configuration dictated the eigenvector of **C** with largest eigenvalue. Depending on the specific form of **C**, the configuration may coincide with the one minimizing the cost function. This means that the PO network deterministically solves the selected optimization problem when driven above the threshold. Such a phenomenology allows to conclude that the optimization problem belongs to the polynomial (P) class of computational complexity^49,68^, because finding the ground state of Eq. (3) reduces to finding the eigenvector of **C** with maximal eigenvalue. This is indeed the case of panels a, e and c, g in Fig. 5 with uniform antiferromagnetic interaction. For general NP problems, the pump amplitude has to be increased to let the system explore a larger configuration space and reduce the heterogeneity of {*S*_*q*_}, to increase the quality of the solution. When the spin variables are discrete (*D* = 1), this results into finding the correct solution of the Ising model with finite success probability^69^.

**Fig. 6:**
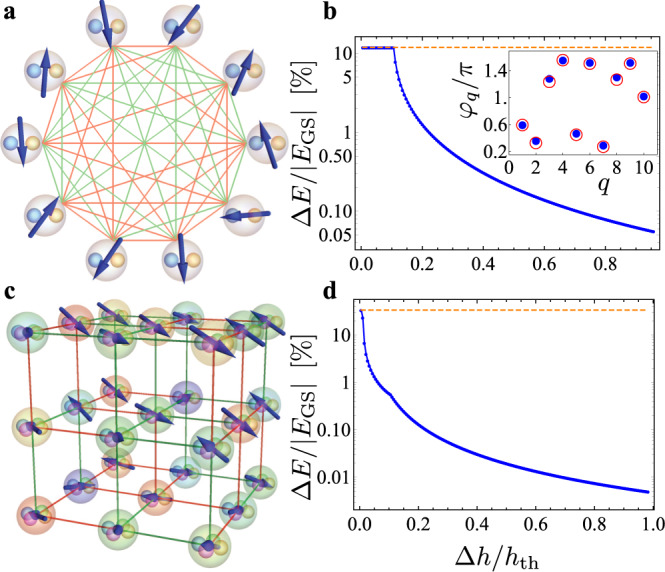
*D*-vector Hamiltonian minimization. Data for **a**, **b** the XY model (*D* = 2) with *N* = 10 and complete random K graph in Fig. 4, and **c**, **d** the QCD spin-glass model (*D* = 4) with *N* = 27 in Fig. 5. Panels **b** and **d** show, respectively, for panels **a** and **c** the spin relative energy deviation (in percentage) Δ*E*/∣*E*_G*S*_∣, where Δ*E* = *E*_P*O*_ − *E*_G*S*_, between the energy *E*_P*O*_ computed from the PO amplitudes and the GS energy *E*_G*S*_ by numerical minimization of the selected XY and QCD Hamiltonian, as a function of the pump amplitude deviation from threshold. The horizontal orange dashed lines mark the spin energy from the eigenvector of **C** with largest eigenvalue. Blue dots and red open circles in the inset of panel **b** are the XY phases *φ*_*q*_ from the POs for Δ*h*/*h*_t*h*_ = 0.8, and the GS phases by numerically minimizing the XY Hamiltonian, respectively. By increasing the pump amplitude, the energy from the POs rapidly approaches the computed GS energy, with determined deviation below 0.1% for the case in panel **b**, and below 0.01% for that in panel **d**.

For the multidimensional hyperspin case *D*≥2, the way the PO network performs the optimization of Eq. (3) is shown in Fig. 6. We focus specifically on the XY model with K graph in Fig. 4b, f, and the random spin glass in Fig. 5d, h with *D* = 4. The phenomenology is common to other choices of **J** (see Supplementary Note 3). We show in panels b, d (blue color) the PO relative energy deviation Δ*E*/∣*E*_GS_∣, where Δ*E* = *E*_PO_ − *E*_GS_, as a function of the pump amplitude deviation from threshold Δ*h* = *h* − *h*_th_. The pump amplitude *h* varies from the analytical threshold to a numerically determined value, above which the PO amplitudes acquire a nonzero imaginary part^50^. The PO energy *E*_PO_ is found from Eq. (3) by determining the hyperspins from the PO steady-state amplitudes ${\overrightarrow{\sigma }}_{q}=({\overline{X}}_{1}^{(q)},\ldots,\,{\overline{X}}_{D}^{(q)})/{S}_{q}$, and the ground-state value *E*_GS_ is found by numerically minimizing Eq. (3) with respect to the real variables $\{{X}_{\mu }^{(q)}\}$ using the minimizer NMinimize in Wolfram Mathematica. The horizontal orange dashed line marks the PO energy of the eigenvector of the coupling matrix with largest eigenvalue. We find that the PO energy deviation from the computed ground-state value starts from the eigenvector value and monotonically decreases as the pump amplitude is increased above threshold. This result is intimately related to the decrease of hyperspin amplitude heterogeneity for increasing pump (see Supplementary Note 2 and 3). For the XY model in panels a, b, we find that the the energy deviation reaches values that are below approximately 0.1%, while for the spin-glass QCD model in panels c, d, the energy deviation goes even below approximately 0.01%. The inset in panel b shows the phases *φ*_*q*_/*π* of the *N* = 10 XY spins, computed from the PO phases (blue filled circles) and from the numerical minimization of the XY Hamiltonian (open red circles), for a pump amplitude Δ*h*/*h*_th_ = 0.8. As evident, the two data series are overlapped. In light of these results, we conclude that our PO network in Eq. (5) finds to a very good approximation the ground-state of the *D*-vector hyperspin model.

### Dimensional annealing

**Fig. 7:**
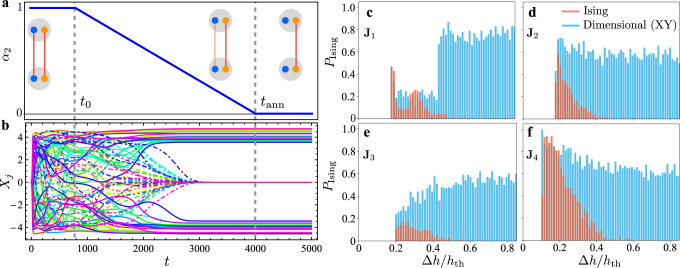
Performance comparison between dimensional annealing and discrete spin simulation. We use *N* = 40 spins and four random K graphs with adjacency matrices **J**_*u*_ with *u* = 1, …, 4. **a** Dependence of *α*_2_ on time *t* during the annealing. The vertical dashed gray lines mark the starting *t*_0_ and final annealing time *t*_ann_. The insets depict the connectivity between any two XY spins (with negative coupling for illustration purposes). Here, *α*_2_ is the metric component along the *y*-axis, which is turned to zero at the end of the annealing. **b** Dynamics of the real PO amplitudes *X*_*j*_ from Eq. (5) with *β* = 10^−2^ during the annealing. After an initial dynamics, the POs corresponding to the *y*-components of the XY spins (dot-dashed lines) switch off, while the *x*-components reach a steady state (full lines, see Supplementary Movie 4). **c**–**f** Histograms of Ising success probability *P*_Ising_ from the discrete spin simulation (*D* = 1, red histograms), and from the simulation of the XY model with annealing (interpolating between *D* = 2 and *D* = 1, blue histograms), as a function of the pump power deviation from threshold Δ*h*/*h*_th_ and for a given **J**_*u*_ as in the labels. As evident, the dimensional annealing significantly increases *P*_Ising_ for a sufficiently large pump amplitude.

A notable advantage of the hyperspin machine compared to state-of-the-art continuous spin simulators is the ability to define a spin according to its Cartesian projections. This opens the possibility to emulate quantum-inspired and adiabatic-computation algorithms to solve optimization problems using a purely classical system. We now discuss one of such remarkable applications, i.e., solving the Ising model by performing an annealing protocol starting from the XY model. This application follows from using a time-dependent diagonal metric tensor **G**(*t*) = diag(*α*_1_, …, *α*_*D*_), where *α*_*μ*_ = *α*_*μ*_(*t*) is a time-dependent metric component. Starting from the XY Hamiltonian [Eq. (3) with *D* = 2] at time *t* = 0, which is for **G** = diag(1, 1), we arrive at the Ising Hamiltonian [Eq. (3) with *D* = 1] for times *t* larger than a given “annealing” time *t*_ann_, with **G** = diag(1, 0). We use *α*_1_ = 1 and independent of *t*. For the other time-dependent metric component, we take *α*_2_(*t*) = 1 for a time *t* smaller than a fixed *t*_0_, which is the starting time of the annealing procedure, and *α*_2_(*t*) = 0 for *t* > *t*_ann_. For an intermediate time between *t*_0_ and *t*_ann_, the metric component linearly interpolates between 1 and 0 (see Fig. 7a). In this way, the PO network simulates *H*_XY_ for a time *t* < *t*_0_, it reduces to *H*_Ising_ for *t* > *t*_ann_, while for *t* between *t*_0_ and *t*_ann_, it interpolates between the two models, i.e., *H*(*s*) = (1 − *s*)*H*_XY_ + *sH*_Ising_, where *s* = (*t* − *t*_0_)/(*t*_ann_ − *t*_0_). The resulting PO amplitude dynamics is shown in panel **b**. During an initial nontrivial dynamics for a time smaller than *t*_0_, the PO network simulates the XY model starting from random initial conditions. In this first stage, as shown in Fig. 6, the system starts to converge towards the minimum of the XY Hamiltonian. For a time larger than *t*_0_, the reduction of the *α*_2_ metric causes the POs corresponding to the *μ* = 2 components (i.e., *y*, dot-dashed lines) to gradually switch off. The other PO amplitudes defining the *μ* = 1 components (i.e., *x*, full lines) converge to a steady-state following a dynamics dominated by the Ising Hamiltonian. After the annealing procedure, the XY spins are polarized along the *x*-axis and represent an Ising state (see Fig. 4). The reason why we start the annealing procedure after a finite time *t*_0_ is to let the PO amplitudes be amplified sufficiently above the initial random values before reducing the system dimensionality. We remark that the annealing protocol proposed here is not a mere emulation of the conventional quantum annealing, where one seeks for the ground state of the classical Ising model with *z*-aligned spins starting from a configuration along an orthogonal direction (*x* or *y*)^70^. Our protocol performs a “dimensional crossover” between two *D*-vector models with the same adjacency matrix **J** but in different dimension, specifically from *D* = 2 to *D* = 1. For this reason, we name our protocol as dimensional annealing.

We now focus on a network of *N* = 40 spins and show that the dimensional annealing dramatically increases the success probability to solve the Ising model. To reach this goal, we proceed as follows. We choose four adjacency matrices **J**_*u*_ with *u* = 1, 2, 3, 4 representing four random complete K graphs with binary edge weights ∣*J*_*qp*_∣ = 0.02. For each adjacency matrix, we repeat the numerical integration of the PO amplitudes equations for *D* = 1 a number *M* = 100 of times, and retrieve for each run the Ising spin values from the steady-state amplitudes as described before. From the obtained phases, the *M* Ising energies ${E}_{m,{{{{{{{\rm{PO}}}}}}}}}^{({{{{{{{\rm{Ising}}}}}}}})}$ are computed, where *m* = 1, …, *M*. The success probability *P*_Ising_ is defined as the number of runs such that ${E}_{m,{{{{{{{\rm{PO}}}}}}}}}^{({{{{{{{\rm{Ising}}}}}}}})}={E}_{{{{{{{{\rm{GS}}}}}}}}}^{({{{{{{{\rm{Ising}}}}}}}})}$, divided by *M*. To find the global Ising ground-state energy ${E}_{{{{{{{{\rm{GS}}}}}}}}}^{({{{{{{{\rm{Ising}}}}}}}})}$, we resort to a Monte-Carlo Metropolis-annealing inspired algorithm^71^. We remark that, differently from the cases *D* ≥ 2 in Fig. 6, we cannot here resort to the numerical minimization of *H*_Ising_ because the minimization is more likely to get stuck in local minima due to discrete nature of the spin variables for *D* = 1. The computation of *P*_Ising_ is performed for different values of the pump amplitude deviation from threshold Δ*h*/*h*_th_ and plotted as red histograms in panels c, d, e, f of Fig. 7. As evident, the success probability is nonzero only in a narrow range of *h* > *h*_th_, and the details of the histograms critically depend on the coupling matrix. These observations are consistent with those in ref. 63. The fact that the PO network does not find the global solution of the Ising Hamiltonian is a signature of the NP-hard nature of the optimization problem. As the pump amplitude increases, the success probability decreases. This fact is ascribed to the heterogeneity of the amplitudes^61^: The PO system explores a larger configurational space, and the probability to converge to the global minimum of the Ising model decreases.

**Fig. 8:**
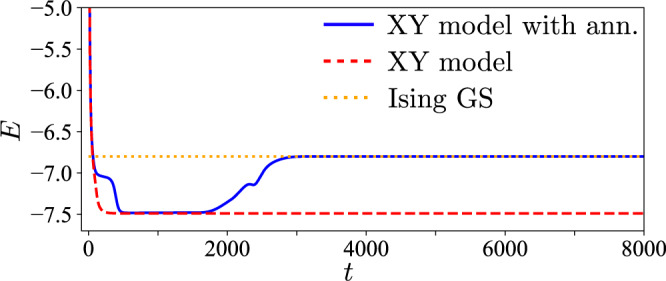
Energy during the dimensional annealing. Dynamics of the spin energy *E*(*t*) from the XY model simulation (*D* = 2, red dashed line) and the XY model with dimensional annealing (from *D* = 2 to *D* = 1, blue line). The horizontal dotted line marks the calculated ground-state energy of the Ising model (*D* = 1). The energy first tends to reach the steady state of the XY model. Then, the dimensional annealing drives the energy to a new steady state of the Ising Hamiltonian, which is the Ising ground state, found at a higher energy compared to the XY steady-state energy.

We then simulate the XY model with dimensional annealing for the same adjacency matrices **J**_*u*_, and compute the success probability of the Ising model (see panel a, b). The resulting histograms are shown in blue in panels c, d, e, f, and compared to the red histograms computed for *D* = 1. While the success probability from the dimensional annealing (blue) and from the discrete spin simulation (red) are comparable within the narrow range of pump amplitude where *P*_Ising_ from the discrete spin simulation is sufficiently large, the success probability for the dimensional annealing stays above 50% even for large pump amplitudes, where the corresponding value from the discrete spin simulation is very small or zero, within our numerical precision. In other words, we find an orders of magnitude increase in the success probability from the *D* = 2 simulation in a parameter range where the conventional *D* = 1 approach fails (including NP-hard cases). This remarkable result is a consequence of the fact that the hyperspin machine finds the state of a discrete spin model from the dynamics of a continuous spin system. At variance with previous studies of high-dimensional spins in the context of semidefinite programming, based on post-processing optimal states in *N* dimensions^72^, the dimensional annealing simulates the dynamics of coupled POs, allowing to gradually reach in time the target discrete model Hamiltonian. This has a twofold advantage in terms of increasing the probability to find the global minimum of the discrete model: First, the Ising ground-state configuration (i.e., the state with all spins oriented along the same direction) is a particular excited state of the larger class of XY states (i.e., spins taking any orientation on the *xy*-plane). This fact is exemplified in Fig. 8, where the time variation of the energy *E* from the XY model with and without dimensional annealing is shown. After a first dynamical transient where the energy tends to the steady-state value of the XY model, the dimensionality reduction drives the energy to a minimum of the Ising Hamiltonian, at higher energy compared to the XY model steady-state value. As such, local minima of the energy landscape can be smoothly escaped by exploiting the additional dimension starting from an energy value that is in general below the target one. In contrast, escaping a local minimum in the discrete model itself is harder since it can occur only by full spin flips, which intrinsically requires to overcome a larger stiffness compared to the continuous case. Second, the additional local minima introduced by the second dimension are adiabatically eliminated in time by the dimensional crossover. Therefore, the final minimum found by the annealing is by construction a minimum of the Ising Hamiltonian. Before concluding, we remark that this mechanism to escape local minima of the discrete target model is profoundly different from that of noisy Ising simulators, where noise is either an additive noise inducing fluctuations of the real PO amplitudes^20,51^, or an effective noise resulting from the fluctuating dynamics of real and imaginary PO amplitudes around a fixed point^21^. In this case, when the Ising spins are defined from the real PO amplitudes, noise simply eases the exploration of the Ising energy landscape by inducing random flips of the Ising spin. In proper conditions, such an erratic exploration prevents the system from being stuck in high-energy states of the Ising Hamiltonian, thus enhancing the probability to reach low-energy minima from states at higher Ising energy. The dimensional annealing, instead, eases the exploration of the energy landscape by tuning the system topology, adiabatically changing the energy landscape from the XY to the Ising one, and allowing the reach of low-energy Ising configurations from states at lower energy.

## Discussion

We propose and theoretically validate a network of coupled POs to simulate systems of hyperspins in general dimension *D*. An isolated hyperspin is realized by feeding *D* POs with the same pump field, forming a PO multiplet, and a network of coupled hyperspins is achieved by coupling POs belonging to different multiplets. Focusing on PO connectivities implementing the standard Euclidian scalar product, we show that our system finds to a very good approximation the minimum of the *D*-vector spin Hamiltonian. An advantage of our proposal is that we construct an hyperspin from its Cartesian coordinates, each represented by a specific PO in the multiplet. Thus, we can implement spin models with arbitrary connectivity and emulate quantum algorithms on a purely classical system. We exploit this feature to propose a dimensional annealing protocol, which interpolates between the XY and Ising Hamiltonians. We show that our protocol significantly enhances the success probability to find the global minimum of the Ising Hamiltonian for selected coupling matrices. Intriguing future developments will be the implementation of effective magnetic fields, whose realization with POs for the Ising model has been proposed in ref. 73, as well as the simulation of nonzero temperature in a controllable way^37^. The hyperspin machine paves the way towards the numerical and experimental study of previously unaccessible critical phenomena in advanced spin models, as well as the simulation of quantum spin models like the Ising model in a transverse field^74^ at an unprecedented scale. In this manuscript, we focus on the *D*-vector spin model, but our system allows the implementation of general spin Hamiltonians where a PO of a given spin is connected to any other PO in another spin, i.e.,

6 $${H}_{{{{{{{{\rm{spin}}}}}}}}}(\{\overrightarrow{\sigma }\})=-\mathop{\sum }\limits_{q,p=1}^{N}{J}_{qp}\mathop{\sum }\limits_{\mu,\nu=1}^{D}{G}_{\mu \nu }\,{\sigma }_{\mu }^{(q)}{\sigma }_{\nu }^{(p)},$$

The hyperspin machine can hence simulate spin models with anisotropic interactions. A relevant case is with *D* = 3, which describes the anisotropic Heisenberg model with symmetric and Dzyaloshinsky-Moriya interactions stabilizing nontrivial magnetic textures in solids^43,75,76^. Furthermore, in this manuscript, we focus on identical PO multiplets. However, the hyperspin machine allows multiplets of any size within the same network, opening the possibility to realize models with hybrid symmetries^77^. The design of the hyperspin machine with POs opens the future perspective to experimentally realize fully optical, scalable, and size-independent continuous spin simulators, extending recent proposals with an optical cavity with a nonlinear medium and spatial light modulators, similar to that in ref. 49 for the Ising model.

## Methods

### Multiple-time scale expansion

The equations of motion for the slow-varying complex PO amplitudes {*X*_*j*_} in Eq. (2) are found from those of the PO variables {*x*_*j*_} in Eq. (1) using the multiple-time scale perturbative method in refs. 50, 52. We identify a small expansion parameter (*g*) and define two different time scales, one for the fast oscillations at frequency *ω*_0_, identified by *t*, and another one *gt* characterizing the slow dynamics of the PO amplitudes. We then expand ${x}_{j}\equiv {x}_{j}(t,\,gt)={x}_{j}^{(0)}+g\,{x}_{j}^{(1)}$. By plugging this expansion in the equations of motion and by separating terms that are multiplied by *g* by those that are not, we first obtain ${x}_{j}^{(0)}(t,\,gt)={X}_{j}(gt){e}^{i{\omega }_{0}t}+{X}_{j}^{*}(gt){e}^{-i{\omega }_{0}t}$, and then by imposing the solvability condition for ${x}_{j}^{(1)}$, i.e., requiring that all terms in the equation ${d}^{2}{x}_{j}^{(1)}/d{t}^{2}$ that are multiplied by ${e}^{\pm i{\omega }_{0}t}$ vanish, the equation of motion for the complex amplitudes {*X*_*j*_} are obtained

7 $$\frac{\partial {X}_{j}}{\partial t}=	 \frac{h}{4}\,{X}_{j}^{*}-\frac{1}{2}\,{X}_{j}\\ 	 -\frac{h\beta }{4}\mathop{\sum }\limits_{l=1}^{D}\left(2{|{X}_{l}|}^{2}{X}_{j}^{*}+{({X}_{l}^{*})}^{2}{X}_{j}-{X}_{l}^{2}{X}_{j}\right)$$

and in the long-time limit, where the imaginary part is exponentially suppressed (see Supplementary Note 1) so ${X}_{j}\in {\mathbb{R}}$, for all *j*, Eq. (7) reduces to Eq. (2).

### Hyperspin network as gradient descendent

The fact that the system in Eq. (5) behaves as a gradient descendent driving the spin configuration towards the minimum of the *D*-vector spin model Hamiltonian in Eq. (3) when amplitude heterogeneity is suppressed, is proved by showing that the dynamics tends to minimize the Lyapunov function

8 $$V({X}_{1},\ldots,\,{X}_{DN})=-\mathop{\sum }\limits_{q=1}^{N}\left[\frac{1}{2}\left(\frac{h}{4}-\frac{g}{2}\right){S}_{q}^{2}-\frac{h\beta }{8}{S}_{q}^{4}\right]\\ -\frac{1}{4}\mathop{\sum }\limits_{q,p=1}^{N}{J}_{qp}\mathop{\sum }\limits_{\mu=1}^{D}\mathop{\sum }\limits_{\nu=1}^{D}{G}_{\mu \nu }{X}_{\mu }^{(q)}{X}_{\nu }^{(p)}.$$

It can be shown by explicit inspection that ∂*V*/∂*X*_*k*_ = − *dX*_*k*_/*dt* (see Supplementary Note 2) and then

9 $$\frac{dV}{dt}=\mathop{\sum }\limits_{k=1}^{DN}\frac{\partial V}{\partial {X}_{k}}\frac{d{X}_{k}}{dt}=-\mathop{\sum }\limits_{k=1}^{DN}{\left(\frac{d{X}_{k}}{dt}\right)}^{2},$$

which means that the dynamics is such that *dV*/*dt* ≤ 0, where the lower bound *dV*/*dt* = 0 is found at the steady state (fixed point) where *dX*_*k*_/*dt* = 0, for all *k*. It follows that the fixed point is a minimum of *V* (i.e., *V* is bounded from below). The fact that *dV*/*dt* < 0 plus the existence of a steady-state allows to conclude that *V* in Eq. (8) is a Lyapunov function for the system in Eq. (5), and the dynamics drives the system towards a minimum of *V*. To relate the minimum of *V* to that of *H*_*D*_ in Eq. (3) when ${{{{{{{\bf{G}}}}}}}}={{\mathbb{1}}}_{D}$ and when amplitude heterogeneity is suppressed, we define $\overline{S}={N}^{-1}\mathop{\sum }\nolimits_{q=1}^{N}{S}_{q}$, and ${S}_{q}=\overline{S}(1+{\delta }_{q})$, where *δ*_*q*_ denotes the deviation of *S*_*q*_ from the equalized amplitude $\overline{S}$. Then ${X}_{\mu }^{(q)}={\sigma }_{\mu }^{(q)}{S}_{q}={\sigma }_{\mu }^{(q)}\overline{S}(1+{\delta }_{q})$. By using this expression of $\{{X}_{\mu }^{(q)}\}$ into Eq. (8), we have

10 $$V({X}_{1},\ldots,\,{X}_{DN})=-N{\overline{S}}^{2}\left[\frac{1}{2}\left(\frac{h}{4}-\frac{g}{2}\right)-\frac{h\beta {\overline{S}}^{2}}{8}\right]\\+\frac{{\overline{S}}^{2}}{4}{H}_{D}+v({X}_{1},\ldots,\,{X}_{DN}),$$

where the correction *v*(*X*_1_, …, *X*_*DN*_) includes terms where at least one power of *δ*_*q*_ appears. When the amplitudes are exactly equalized, i.e., *δ*_*q*_ = 0, for all *q*, so *v* = 0, the dynamics tends towards a minimum of a function that is proportional to *H*_*D*_, apart from a constant shift and a rescaling by the positive quantity ${\overline{S}}^{2}/4$. For small *δ*_*q*_, the minima of *V* deviate from those of *H*_*D*_, but the deviation is of order of ${\max }_{q}\{|{\delta }_{q}|\}$, and so the PO amplitude configuration corresponding to the minimum of *V* is expected to be close to that of *H*_*D*_, which is, the ground-state hyperspin configuration. This is indeed the scenario found in our numerical simulations, i.e., the fact that the energy from the hyperspin machine approaches the ground-state energy of *H*_*D*_ (see Fig. 6) is intimately related to the reduction of amplitude heterogeneity, quantified by the heterogeneity degree ${A}_{{{{{{{{\rm{het}}}}}}}}}:={\max }_{q}\{{\delta }_{q}\}-{\min }_{q}\{{\delta }_{q}\}$ (see Supplementary Note 3).

### Details on the numerical simulations

In the numerical data on the energy minimization in Fig. 6, the value of *E*_PO_ is computed as the minimal spin energy retrieved from the steady-state PO amplitudes out of 50 repetitions of the PO network dynamics, for fixed simulation parameters. This is done to avoid detecting energy values of excited states, which in general may happen if the PO dynamics converges to local minima of the energy landscape. Notice that the reduction of amplitude heterogeneity ensures only that the mapping between the PO network cost function and the target cost function (coupled spins) is proper, but in general it does not ensure the convergence to the ground-state solution, since local minima of the spin Hamiltonian may always be encountered^61^. In our numerics, we checked that the retrieved spin configuration indeed corresponded to a global minimum (no value lower than the reported values of *E*_PO_ was ever detected). The value of *E*_GS_ is computed by minimizing the *D*-vector spin Hamiltonian for the same adjacency matrix by using NMinimize in Wolfram Mathematica. Our numerical code to find the steady-state PO amplitudes performs the integration of Eq. (5) in C language by means of a fourth-order Runge-Kutta method with time step 0.05, and the results are double checked by integrating the same equations by the numerical integrator NDSolve in Wolfram Mathematica.

## Supplementary information

Supplementary Information

Description of Additional Supplementary Files

Supplementary Movie 1

Supplementary Movie 2

Supplementary Movie 3

Supplementary Movie 4

## Data Availability

All the data supporting the findings in this work are available within the manuscript and Supplementary Information files, and any additional data are available from the corresponding author upon reasonable request.
