# Supplementary material for: Multidimensional hyperspin machine
Source: Nat Commun. 2022 Nov 25;13:7248. doi: 10.1038/s41467-022-34847-9 (PMC9700766; doi:10.1038/s41467-022-34847-9)
Supplement: Supplementary file 1 — Supplementary Information [file 41467_2022_34847_MOESM1_ESM.pdf]

Supplementary information: Multidimensional hyperspin machine

Marcello Calvanese Strinati[1,2] and Claudio Conti[3,2,1]
[1]Centro Ricerche Enrico Fermi (CREF), Via Panisperna 89a, 00184 Rome, Italy
[2]Institute for Complex Systems, National Research Council (ISC-CNR), 00185 Rome, Italy
[3]Physics Department, Sapienza University of Rome, 00185 Rome, Italy
(Dated: November 4, 2022)

Supplementary Note 1. Equations of motion for the complex PO amplitudes

We here report the derivation of the equations of motion for the complex parametric oscillator (PO) amplitudes. Different POs are coupled by a linear dissipative coupling described by a symmetric $(D \times N) \times (D \times N)$ matrix $\mathbf{C}$, whose (j, l)-th entry quantifies the dissipative coupling between x_j and x_l. The system is described by the $D \times N$ coupled classical Mathieu's equations as

$$\ddot{x}_j + \omega_0^2 \left[1 + gh\left(1 - \beta \sum_{l=1}^{DN} W_{jl} x_l^2\right) \sin(2\omega_0 t)\right] x_j + \omega_0\, g\, \dot{x}_j - \omega_0 g \sum_{l \neq j} C_{jl}\, \dot{x}_l = 0 \qquad (j = 1, \ldots, DN)\ . \tag{1}$$

The $(D \times N) \times (D \times N)$ matrix $\mathbf{W}$ is a block diagonal matrix given by $\mathbf{W} = \mathbb{1}_N \otimes \mathcal{I}_D$, where "$\otimes$" denotes the Krönecker product, $\mathbb{1}_N$ is the $N \times N$ identity matrix, and $\mathcal{I}_D$ is the $D \times D$ matrix with all entries equal to 1. This matrix expresses the nonlinear coupling between POs induced by common pump saturation, which is responsible for the arrangement of the $D \times N$ POs as N multiplets D POs each. The matrix $\mathbf{C}$ defines the linear coupling between POs. When chosen of the form $\mathbf{C} = \mathbf{J} \otimes \mathbf{G}$, where $\mathbf{J}$ is the $N \times N$ adjacency matrix with zero diagonal entries, and $\mathbf{G}$ is the $D \times D$ metric tensor (see main text), this linear coupling couples POs in different multiplets. The nontrivial interplay of nonlinear and linear coupling during the system dynamics realizes the hyperspin machine, as detailed below in Supplementary Note 2.

From Eq. (1), we find the equations of motion for the slow-varying complex amplitudes $\{X_j\}$ of the POs using the multiple time scale perturbative method described in Refs. [1, 2], to which the interested reader is addressed for more details. We here report the main steps for the sake of completeness. For each oscillator x_j, we identify a small expansion parameter (in our case g) and separate the dynamics of the fast oscillations at frequency ω_0 from the dynamics of the complex amplitude X_j occurring on much larger characteristic time scales. These two distinct times are quantified by the time variables t and $\tau = gt$, i.e., we write $x_j = x_j(t, \tau)$, which is further expanded as $x_j = x_j^{(0)} + g\, x_j^{(1)}$. By separating in Eq. (1) terms that are multiplied by g from those that are not, one can write $x_j^{(0)}(t, \tau) = X_j(\tau) e^{i\omega_0 t} + X_j^*(\tau) e^{-i\omega_0 t}$. By using this expression for $x_j(t)$ in Eq. (1) and by imposing the solvability condition for $x_j^{(1)}$, we find the $D \times N$ equations for the complex amplitudes ($\tilde{\tau} = \omega_0 \tau$)

$$\frac{\partial X_j}{\partial \tilde{\tau}} = \frac{h}{4} X_j^* - \frac{1}{2} X_j + \frac{1}{2} \sum_{l=1}^{DN} \left[-\frac{h\beta}{2} W_{jl} \left(2|X_l|^2 X_j^* + (X_l^*)^2 X_j - X_l^2 X_j\right) + C_{jl} X_l\right]\ . \tag{2}$$

By separating the amplitude real and imaginary parts, respectively $X_{j,R} \equiv \mathrm{Re}[X_j]$ and $X_{j,I} \equiv \mathrm{Im}[X_j]$, Eq. (2) can be rewritten as (in units such that $\omega_0 = 1$)

$$\frac{\partial X_{j,R}}{\partial \tau} = \left(\frac{h}{4} - \frac{1}{2}\right) X_{j,R} + \frac{1}{2} \sum_{l=1}^{DN} \left[-h\beta W_{jl} \left[\left(X_{l,R}^2 + X_{l,I}^2\right) X_{j,R} + 2X_{l,R} X_{l,I} X_{j,I}\right] + C_{jl} X_{l,R}\right] \tag{3a}$$

$$\frac{\partial X_{j,I}}{\partial \tau} = \left(-\frac{h}{4} - \frac{1}{2}\right) X_{j,I} + \frac{1}{2} \sum_{l=1}^{DN} \left[h\beta W_{jl} \left[\left(X_{l,R}^2 + X_{l,I}^2\right) X_{j,I} + 2X_{l,R} X_{l,I} X_{j,R}\right] + C_{jl} X_{l,I}\right]\ . \tag{3b}$$

For a not too large pump amplitude h above the oscillation threshold value h_{th}, the dynamics in Eq. (3) amplifies the real parts and suppresses the imaginary parts ($\lim_{\tau \to \infty} X_{j,I} = 0$, for all j). We recall that the equations of motion for the single hyperspin are obtained from Eq. (3) by setting $N = 1$, i.e., $\mathbf{W} = \mathcal{I}_D$ and $\mathbf{C} = 0$. When the imaginary part of the PO amplitudes is zero, the real PO amplitudes evolve according to

$$\frac{\partial X_{j,R}}{\partial \tau} = \left(\frac{h}{4} - \frac{1}{2} - \frac{h\beta}{2} \sum_{l=1}^{D} X_{l,R}^2\right) X_{j,R}\ . \tag{4}$$

As said in the main text, the steady state $\{\overline{X}_{j,R}\}_{j=1}^{D}$ of the single hyperspin, found by equating Eq. (4) to zero, is such that the steady-state values of the real PO amplitudes $\{\overline{X}_{j,R}\}_{j=1}^{D}$ lie on the surface of a D-dimensional hypersphere of radius $S = \sqrt{(1/2 - 1/h)/\beta}$, and $\overline{X}_{j,R}$ defines the j-th Cartesian coordinate of the D-dimensional hyperspin.

For a given coupling matrix $\mathbf{C}$, the oscillation threshold value h_{th} is found by requiring that at least one global mode in Eq. (2) is amplified in time. When the system is close to threshold, as said in Eq. (3), only the real parts of the amplitudes are amplified. In addition, the amplitudes $\{|X_j|\}$ are so small that nonlinearities can be neglected, i.e., one can effectively set $\beta = 0$. By defining $\mathbf{X} = (X_1, \ldots, X_{DN})$, one can write Eq. (2) in the vector form

$$\frac{\partial}{\partial \tau}\mathbf{X} = \left(\frac{h}{4} - \frac{1}{2}\right)\mathbf{X} + \frac{1}{2}\mathbf{C}\mathbf{X} \ , \tag{5}$$

where it is understood that the amplitudes are real. By diagonalizing the coupling matrix as $\mathbf{C} = \mathbf{U}^{\dagger}\mathbf{\Lambda}\mathbf{U}$, where $\mathbf{\Lambda} = \text{diag}(\lambda_1, \ldots, \lambda_{DN})$ and $\{\lambda_j\}$ are the eigenvalues of $\mathbf{C}$, and defining $\mathbf{Y} = \mathbf{U}\mathbf{X}$, one has the diagonal form of Eq. (5)

$$\frac{\partial}{\partial \tau}\mathbf{Y} = \left(\frac{h}{4} - \frac{1}{2}\right)\mathbf{Y} + \frac{1}{2}\mathbf{\Lambda}\mathbf{Y} \ , \tag{6}$$

which is

$$\frac{\partial}{\partial \tau}Y_j = \left(\frac{h}{4} - \frac{1}{2} + \frac{\lambda_j}{2}\right)Y_j \ . \tag{7}$$

From Eq. (7), amplification for the j-th mode is found if

$$\frac{h}{4} - \frac{1}{2} + \frac{\lambda_j}{2} \geq 0 \ , \tag{8}$$

where the lower bound defines the value of the oscillation threshold $h_{j,\text{th}}$ for the j-th mode: $h_{j,\text{th}} = 2\,(1 - \lambda_j)$. The global threshold is the minimal one, i.e., $h_{\text{th}} := \min_j\{h_{j,\text{th}}\}$. By defining the largest eigenvalue as $\lambda_{\max} := \max_j\{\lambda_j\}$, one has

$$h_{\text{th}} = 2\,(1 - \lambda_{\max}) \ . \tag{9}$$

When $\{\lambda_j\}$ are complex numbers, the amplification rate is quantified by the real part of the eigenvalues, i.e., $h_{\text{th}} = 2(1 - \text{Re}[\lambda_{\max}])$, so that $\lambda_{\max}$ is defined as the eigenvalue of the coupling matrix with largest real part.

An example of the PO quadrature dynamics is shown in Supplementary Fig. 1. Panels **a,b,c,d** show the hyperspin network configuration for the random complete K graph with $N = 10$ and $D = 1, 2, 3, 4$ discussed in the main text. The hyperspin configuration is retrieved from the steady state of the PO quadrature dynamics, which is shown in **e,f,g,h** and **i,l,m,n** for the real and imaginary parts, respectively. Starting from initial conditions at time $t = 0$ such that $X_{j,R}(0)$ and $X_{j,I}(0)$ are real random numbers between -0.1 and 0.1 taken from a uniform distribution, the real parts are amplified and eventually reach a steady state that determines the hyperspin state, while the imaginary parts are exponentially suppressed in time for a not too large value of pump amplitude [2], which is the regime of interest in our work.

Supplementary Note 2. Parametric oscillator networks as hyperspin Hamiltonian simulators

We here explicitly derive the connection between the dynamics in Eq. (2), for the case of real amplitudes, and the hyperspin Hamiltonian

$$H_{\text{spin}} = -\sum_{q,p=1}^{N} J_{qp}\, G\left(\vec{\sigma}_q, \vec{\sigma}_p\right) = -\sum_{q,p=1}^{N} J_{qp} \sum_{\mu,\nu=1}^{D} G_{\mu\nu}\, \sigma_{\mu}^{(q)} \sigma_{\nu}^{(p)} \ , \tag{10}$$

with adjacency matrix $\mathbf{J}$ and general metric tensor $\mathbf{G}$.

A. System dynamics and Hamiltonian minimization

Let $j \in \mathbb{S}_q$, where $\mathbb{S}_q := \{j : j = 1 + (q-1)D, \ldots, qD\}$ is the set of indexes defining the q-th PO multiplet (hyperspin). The real amplitude dynamics in Eq. (2) reads (we rename the time variable as t)

$$\frac{dX_j}{dt} = \left(\frac{h}{4} - \frac{g}{2} - \frac{h\beta}{2}\sum_{l=1}^{DN} W_{jl} X_l^2\right) X_j + \frac{1}{2}\sum_{l=1}^{DN} C_{jl} X_l \qquad (j = 1, \ldots, DN) \ . \tag{11}$$

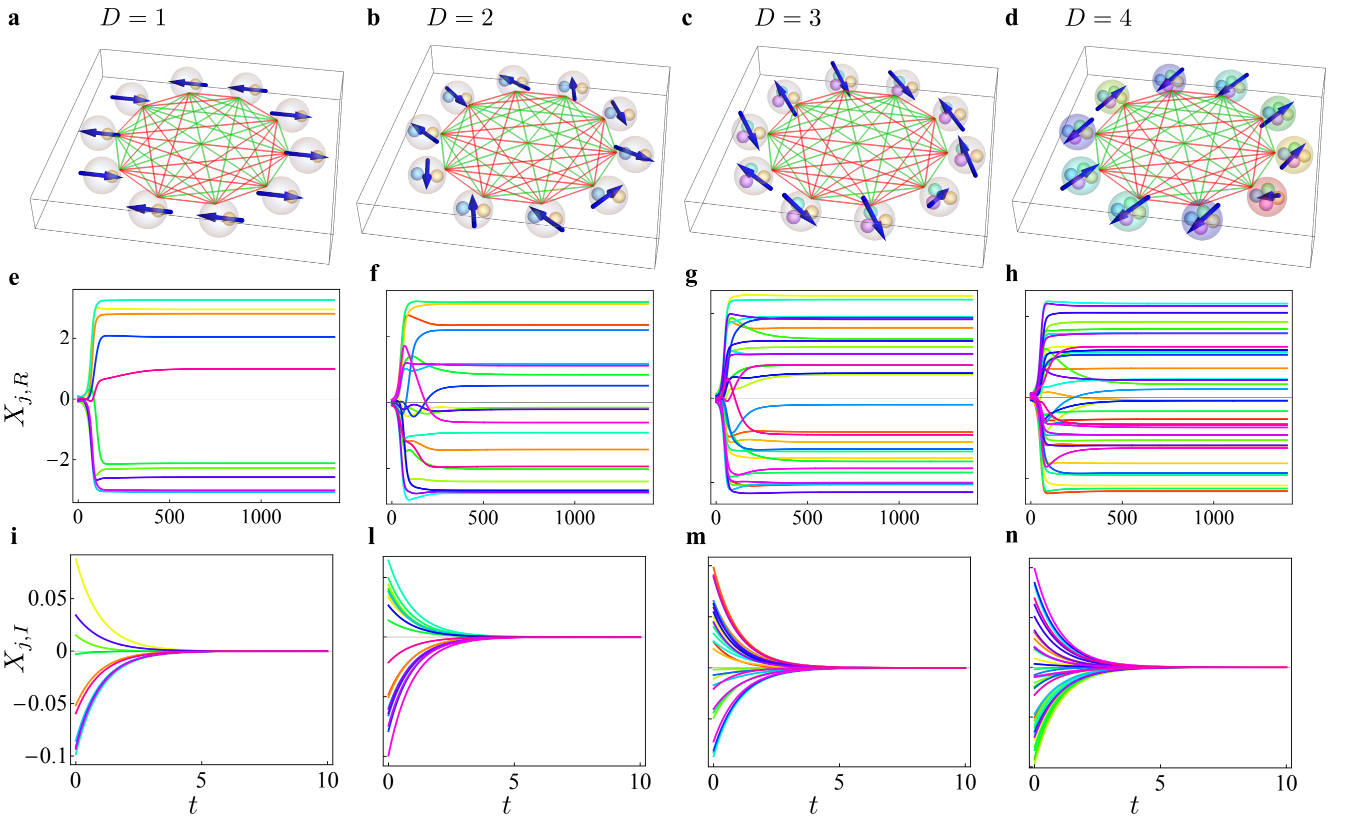

Supplementary Fig. 1. **a,b,c,d** Hyperspin network for different $D = 1, 2, 3, 4$ as in the legends, for the case of $N = 10$ discussed in the main text. **e,f,g,h** Dynamics of the real part of the PO quadratures $X_{j,R}(t)$, and **i,l,m,n** Dynamics of the imaginary part of the PO quadratures $X_{j,I}(t)$. The real parts are amplified starting from random initial conditions, and eventually reach a steady state, which determines the spin configuration in panels **a,b,c,d**, while the imaginary parts are exponentially suppressed in time.

To show that the hyperspin machine minimizes the correct cost function in proper conditions, we determine a global Lyapunov function $V = V(X_1, \dots, X_{DN})$ such that V is bounded from below, i.e., there exists an extremal point $\{\overline{X}_j\}$ such that at this point $dV/dt = 0$, and $dV/dt < 0$ away from the extremal point [3]. We define

$$V(X_1, \dots, X_{DN}) = -\sum_{j=1}^{DN}\left[\frac{1}{2}\left(\frac{h}{4} - \frac{g}{2}\right)X_j^2 - \frac{h\beta}{8}\left(\sum_{l=1}^{DN} W_{jl} X_l^2\right)X_j^2 + \frac{1}{4}\sum_{l=1}^{DN} C_{jl} X_l X_j\right] . \tag{12}$$

and show that Eq. (12) is a Lyapunov function for the system in Eq. (11). The summation of the quartic term in Eq. (12), using the form of $\mathbf{W}$, can be rearranged as

$$\begin{aligned}\sum_{j=1}^{DN}\sum_{l=1}^{DN} W_{jl} X_l^2 X_j^2 = \sum_{j=1}^{DN} X_j^2 \sum_{l=1}^{DN} W_{jl} X_l^2 &= X_1^2 \sum_{l\in\mathbb{S}_1} X_l^2 + X_2^2 \sum_{l\in\mathbb{S}_1} X_l^2 + \ldots + X_D^2 \sum_{l\in\mathbb{S}_1} X_l^2 \\ &\quad + X_{D+1}^2 \sum_{l\in\mathbb{S}_2} X_l^2 + X_{D+2}^2 \sum_{l\in\mathbb{S}_2} X_l^2 + \ldots + X_{2D}^2 \sum_{l\in\mathbb{S}_2} X_l^2 \\ &\quad + \ldots \\ &= \sum_{q=1}^{N}\left(\sum_{l\in\mathbb{S}_q} X_l^2\right)^2 = \sum_{q=1}^{N} S_q^4 \,,\end{aligned} \tag{13}$$

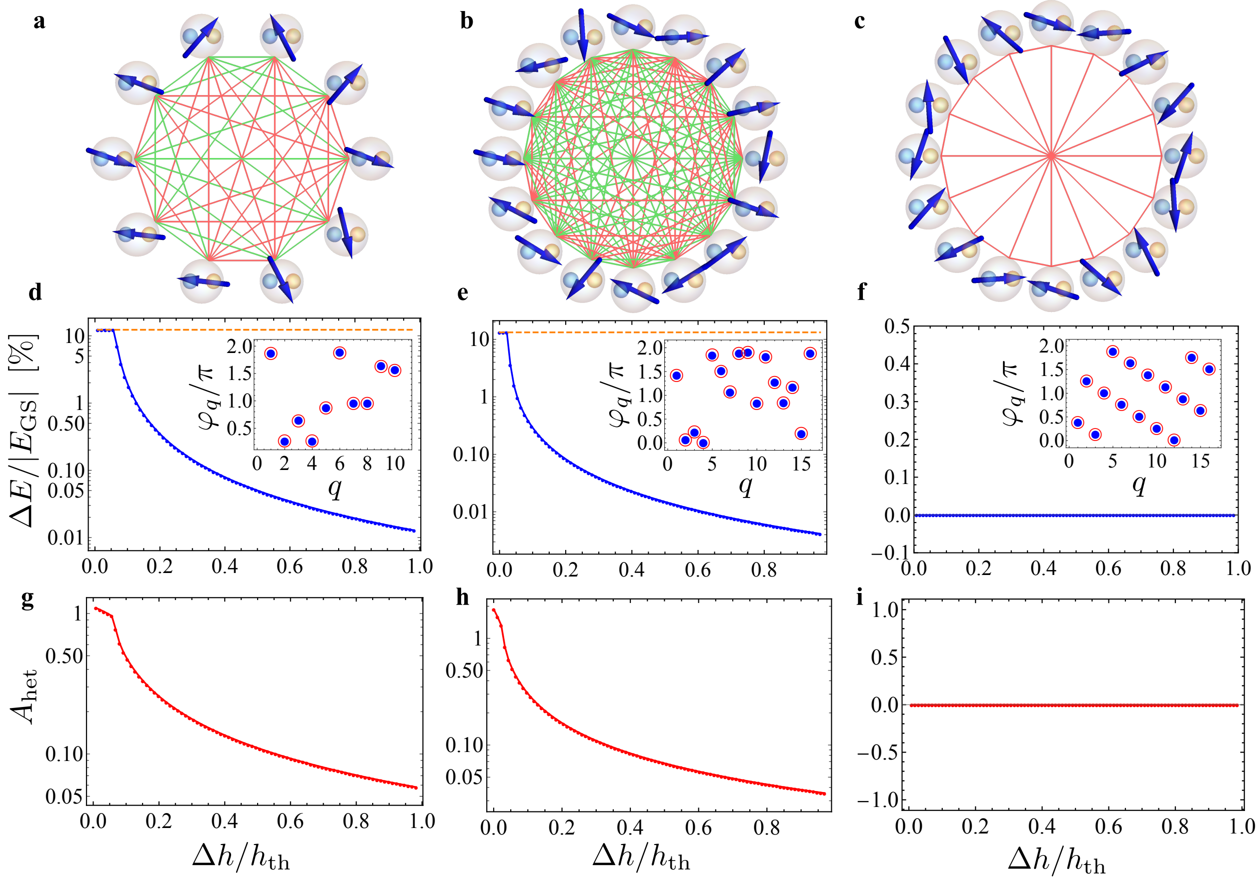

Supplementary Fig. 2. Energy minimization of the D-vector spin Hamiltonian with $D = 2$ (XY model) for **a,d,g** random K graph with $N = 10$, **b,e,h** random K graph with $N = 16$, and **c,f,i** Möbius ladder antiferromagnetic graph with $N = 16$. Panels **a**-**c** show the hyperspin network, panels **d**-**f** the relative energy deviation as a function of pump amplitude deviation from threshold $\Delta h/h_{\rm th}$, and panels **g**-**i** the degree of heterogeneity as a function of $\Delta h/h_{\rm th}$ (notice the log-linear scale for all plots apart from panels **f,i** and insets). The insets in panels **d**-**f** show the XY phases from the PO phases (blue dots) and the computed phases from numerically minimizing the XY Hamiltonian (red circles).

where $S_q^2 = \sum_{l\in\mathbb{S}_q} X_l^2$. When taking the derivative with respect to X_k, where $k \in \mathbb{S}_p$ belongs to the p-th hyperspin, one has

$$\frac{\partial}{\partial X_k}\sum_{j=1}^{DN}\sum_{l=1}^{DN} W_{jl}X_l^2X_j^2 = \frac{\partial}{\partial X_k}\sum_{q=1}^{N}\left(\sum_{l\in\mathbb{S}_q} X_l^2\right)^2 = \frac{\partial}{\partial X_k}\left(\sum_{l\in\mathbb{S}_p} X_l^2\right)^2 = 4X_k\left(\sum_{l\in\mathbb{S}_p} X_l^2\right) , \tag{14}$$

and then by deriving Eq. (12) with respect to X_k one has

$$\frac{\partial V}{\partial X_k} = -\left[\left(\frac{h}{4}-\frac{g}{2}\right)X_k - \frac{h\beta}{2}\left(\sum_{l\in\mathbb{S}_p} X_l^2\right)X_k + \frac{1}{2}\sum_{l=1}^{DN} C_{kl}X_l\right] \equiv -\frac{dX_k}{dt} , \tag{15}$$

and then it follows that

$$\frac{dV}{dt} = \sum_{k=1}^{DN}\frac{\partial V}{\partial X_k}\frac{dX_k}{dt} = -\sum_{k=1}^{DN}\left(\frac{dX_k}{dt}\right)^2 , \tag{16}$$

and indeed one has $dV/dt \le 0$, where the lower bound is found at equilibrium where $dX_k/dt = 0$, for all k (fixed point of the dynamics). Notice that Eq. (12), with the rearrangements of indexes and the explicit form of $\mathbf{C}$ as a Krönecker

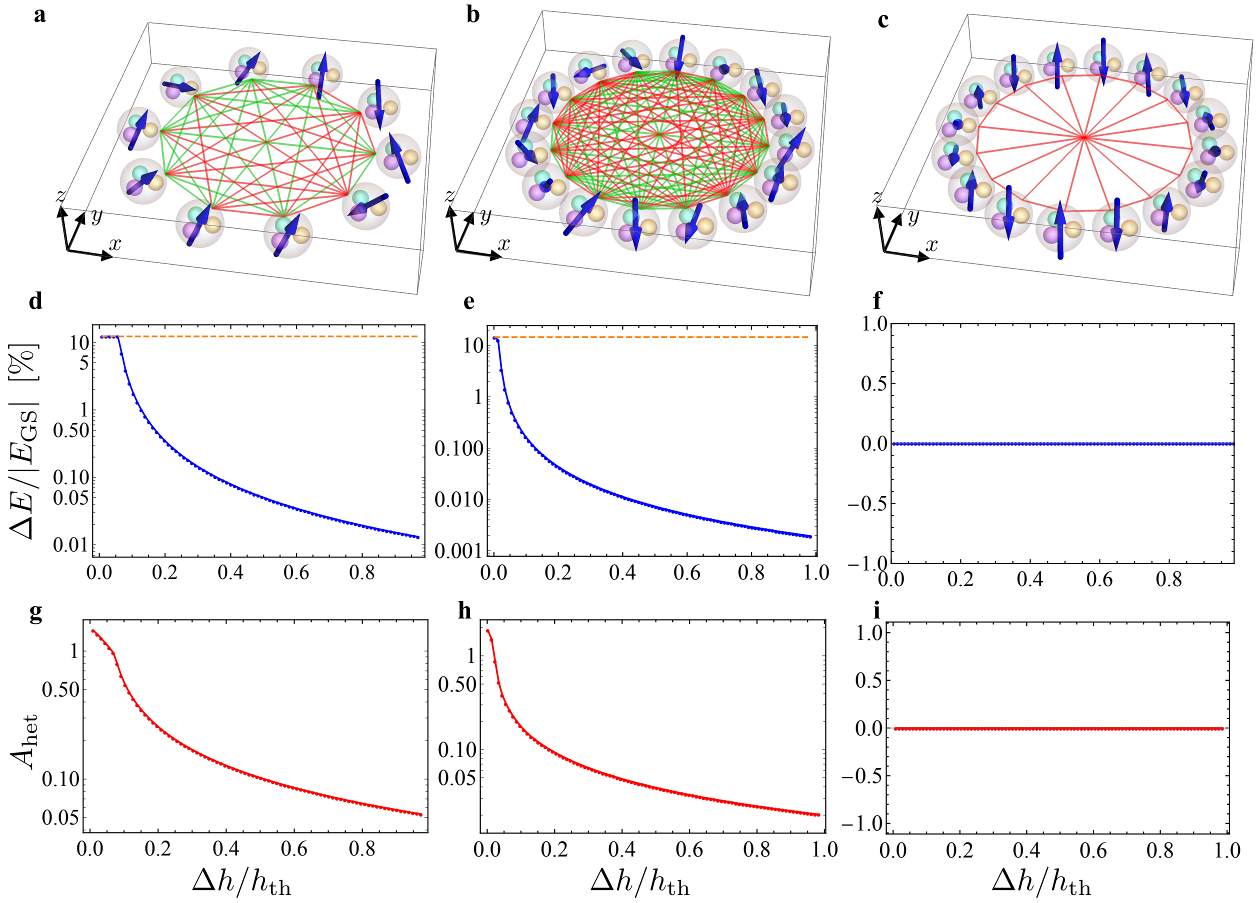

Supplementary Fig. 3. Energy minimization of the D-vector spin Hamiltonian, as in Supplementary Fig. 2 but here with $D = 3$ (Heisenberg model).

product $\mathbf{C} = \mathbf{J} \otimes \mathbf{G}$, can be rewritten as

$$V(X_1, \ldots, X_{DN}) = -\left\{ \sum_{q=1}^{N} \left[\frac{1}{2}\left(\frac{h}{4} - \frac{g}{2}\right) S_q^2 - \frac{h\beta}{8} S_q^4 \right] + \frac{1}{4} \sum_{q,p=1}^{N} J_{qp} \sum_{\mu=1}^{D} \sum_{\nu=1}^{D} G_{\mu\nu} X_\mu^{(q)} X_\nu^{(p)} \right\} , \tag{17}$$

where for the quadratic term (proportional to S_q^2) it is just a rearrangement of indexes, for the quartic term (proportional to S_q^4) one uses Eq. (13), and for the coupling term one proceeds as follows. Let us recall that, for two square matrices $\mathbf{A}$ and $\mathbf{B}$, and for $\mathbf{C} = \mathbf{A} \otimes \mathbf{B}$, the elements of $\mathbf{C}$ are related to the elements of $\mathbf{A}$ and $\mathbf{B}$ as

$$C_{ij} = A_{f(i),f(j)} B_{m(i),m(j)} \qquad f(x) = 1 + \left\lfloor \frac{x-1}{\dim(\mathbf{B})} \right\rfloor \qquad m(x) = 1 + (x-1)\mathrm{mod}[\dim(\mathbf{B})] , \tag{18}$$

where $\dim(\mathbf{B})$ is the dimension of the matrix $\mathbf{B}$, and $\lfloor \cdot \rfloor$ and $\mathrm{mod}(\cdot)$ are the floor and the modulo functions, respectively. Notice that

$$\sum_{j=1}^{\dim(\mathbf{A})\dim(\mathbf{B})} C_{ij} = \sum_{p=1}^{\dim(\mathbf{A})} A_{f(i),p} \sum_{\nu=1}^{\dim(\mathbf{B})} B_{m(i),\nu} . \tag{19}$$

Also, if $\mathbf{v}$ is a vector of dimension $\dim(\mathbf{A})\dim(\mathbf{B})$, where $\dim(\mathbf{A})$ is the dimension of the matrix $\mathbf{A}$, one has

$$\sum_{j=1}^{\dim(\mathbf{A})\dim(\mathbf{B})} C_{ij} v_j = \sum_{p=1}^{\dim(\mathbf{A})} A_{f(i),p} \sum_{\nu=1}^{\dim(\mathbf{B})} B_{m(i),\nu} v_{\nu+(p-1)\dim(\mathbf{B})} . \tag{20}$$

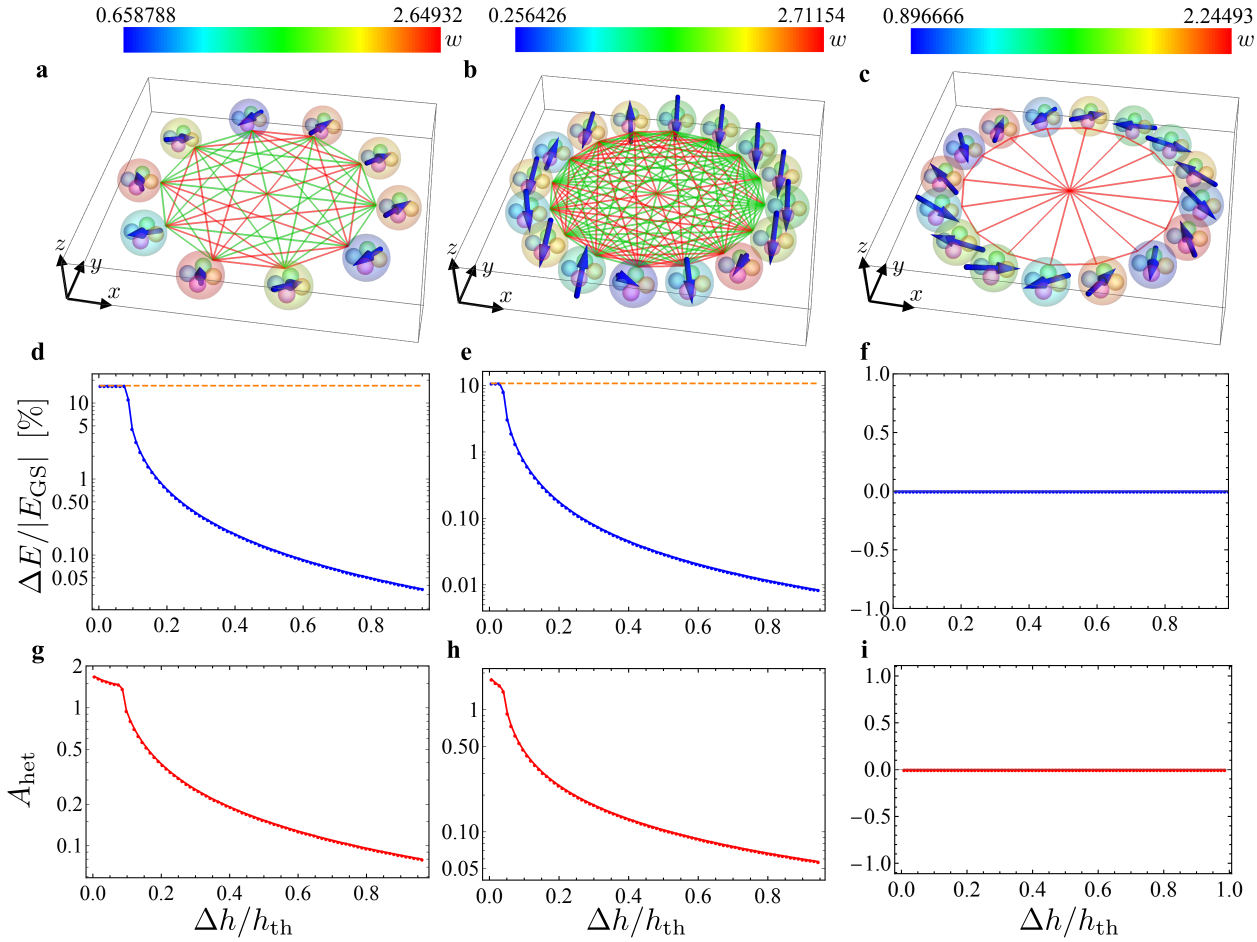

Supplementary Fig. 4. Energy minimization of the D-vector spin Hamiltonian, as in Supplementary Fig. 2 but here with $D = 4$ (QCD model).

By using the fact that $j \in \mathbb{S}_q$ and $l \in \mathbb{S}_p$, and μ and ν label the component of the q-th and p-th hyperspin $\vec{S}_q$ and $\vec{S}_p$, respectively, one can write the coupling term in Eq. (12) as

$$\sum_{j,l=1}^{DN} C_{jl} X_l X_j = \sum_{j,l=1}^{DN} J_{f(j),f(l)}\, G_{m(j),m(l)} X_l X_j = \sum_{q,p=1}^{N} J_{qp} \sum_{\mu,\nu=1}^{D} G_{\mu\nu} X_{\nu+(p-1)D} X_{\mu+(q-1)D}$$

$$= \sum_{q,p=1}^{N} J_{qp} \sum_{\mu=1}^{D}\sum_{\nu=1}^{D} G_{\mu\nu} X_\mu^{(q)} X_\nu^{(p)} = \sum_{q,p=1}^{N} J_{qp}\, G\left(\vec{S}_q, \vec{S}_p\right) \ , \tag{21}$$

where $G(\vec{S}_q, \vec{S}_p) = \sum_{\mu,\nu=1}^{D} G_{\mu\nu} X_\mu^{(q)} X_\nu^{(p)}$. Recall that $\vec{S}_q$ is the vector whose μ-th element is $X_\mu^{(q)}$. Thus, Eq. (17) is just a formal rewriting of Eq. (12) using the definitions of $\mathbf{W}$ and $\mathbf{C}$ given above, and the redefinition of the parametric oscillator indexes. Notice that the fact that a fixed point of the dynamics exists means that the amplitudes $\{X_j\}$, and consequently $\{S_q\}$, do not grow indefinitely. Physically this is possible thanks to the saturation term in Eq. (17) proportional to βS_q^4, which becomes dominant as the amplitude grow and reach the steady state. Since the dynamics is such that $dV/dt < 0$ [gradient descendent, see Eq. (16)], and a steady state (i.e., a fixed point, where $dV/dt = 0$) exists and it is eventually reached by the dynamics (numerically confirmed), it follows that the fixed point is a minimum of V (i.e., V is bounded from below). The fact that $dV/dt < 0$ plus the existence of a steady state allows to conclude that V in Eq. (17) [and of course also in Eq. (12)] is a Lyapunov function for the system in Eq. (11), and the dynamics drives the system towards a minimum of V.

We now explicit the connection between the Lyapunov function and the hyperspin Hamiltonian, when amplitude heterogeneity is reduced. Let us write the average hyperspin amplitude $\overline{S} = N^{-1}\sum_{q=1}^{N} S_q$, and let us define $S_q = \overline{S}(1+\delta_q)$, where δ_q denotes the deviation of S_q from the equalized amplitude $\overline{S}$. With this definition, the amplitudes

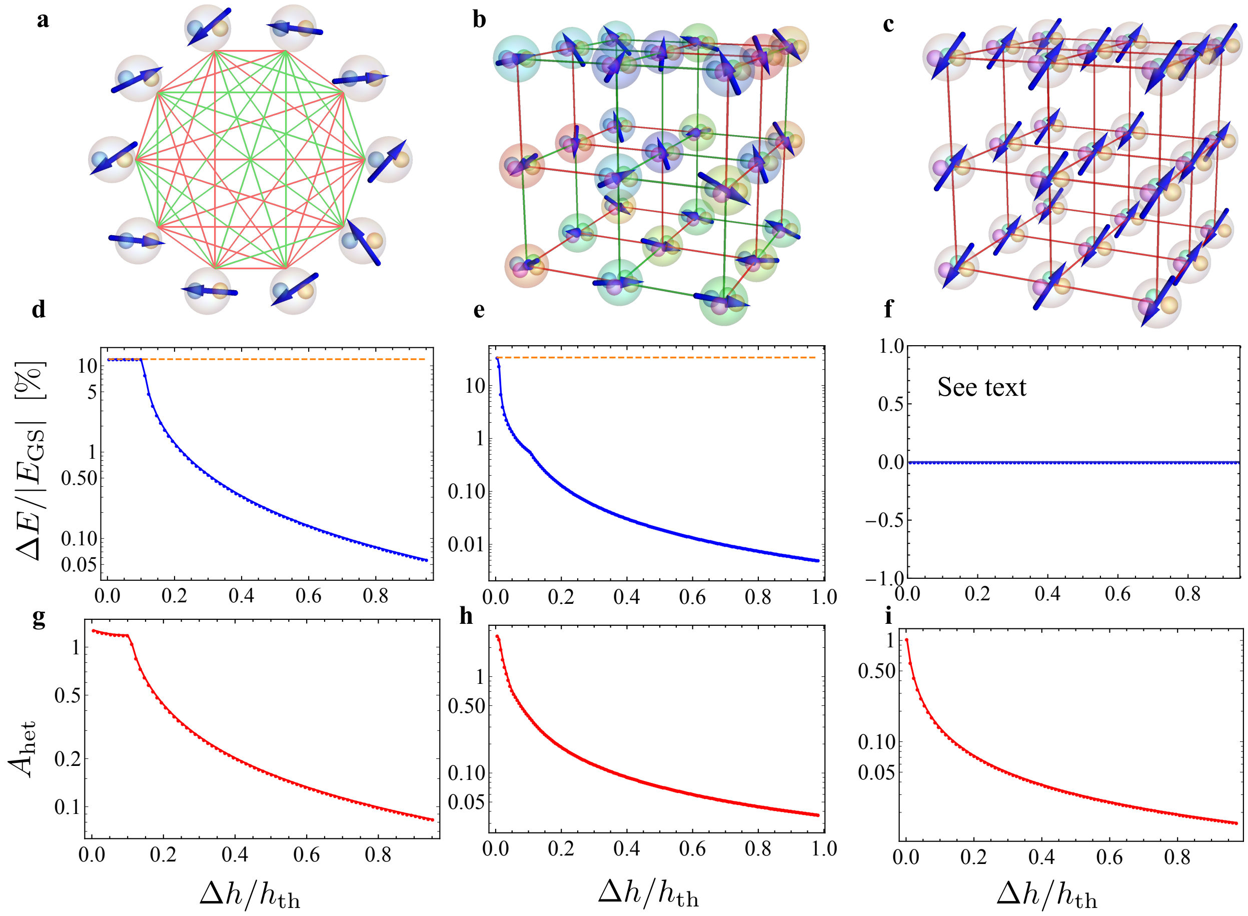

Supplementary Fig. 5. Energy minimization of the D-vector spin Hamiltonian for the cases considered in the main text. **a,d,g**, XY model with random K graph in Fig. 6**a,b** of the main text. **b,e,h**, QCD model with nearest-neighbor 3D solid connectivity and binary interaction in Fig. 6**c,d** of the main text. **c,f,i**, Heisenberg model with solid antiferromagnetic connectivity in Fig. 5**a,e** of the main text. The hard graphs in panels **a,d,g** and **b,e,h** obey the same phenomenology described in Eq. (24) and shown in Supplementary Figs. 2-4, i.e., the reduction of amplitude heterogeneity enhances the quality of the solution by the hyperspin machine. The easy graph in **c,f,i** is one of the special cases of P problem with boundaries (see Supplementary Note 3), where amplitudes are heterogeneous but the hyperspin machine finds the exact ground-state solution at threshold and above.

are equalized when $\delta_q = 0$, for all q. Since $\sigma_\mu^{(q)} = X_\mu^{(q)}/S_q$, one can write $X_\mu^{(q)} = \sigma_\mu^{(q)} S_q = \sigma_\mu^{(q)} \overline{S}(1+\delta_q)$ and then Eq. (17) is written as

$$V(X_1,\ldots,X_{DN}) = -\left\{ \overline{S}^2 \sum_{q=1}^{N} \left[\frac{1}{2}\left(\frac{h}{4} - \frac{g}{2}\right) - \frac{h\beta \overline{S}^2}{8}(1+\delta_q)^2 \right](1+\delta_q)^2 + \frac{\overline{S}^2}{4} \sum_{q,p=1}^{N} J_{qp} \sum_{\mu=1}^{D}\sum_{\nu=1}^{D} G_{\mu\nu}\sigma_\mu^{(q)}\sigma_\nu^{(p)}(1+\delta_q)(1+\delta_p) \right\} . \tag{22}$$

If $\delta_q = 0$, for all q, the amplitudes are equalized and one can write Eq. (22) as

$$\begin{aligned} V(X_1,\ldots,X_{DN}) &= -\left\{ N\overline{S}^2 \left[\frac{1}{2}\left(\frac{h}{4} - \frac{g}{2}\right) - \frac{h\beta\overline{S}^2}{8} \right] + \frac{\overline{S}^2}{4} \sum_{q,p=1}^{N} J_{qp} \sum_{\mu=1}^{D}\sum_{\nu=1}^{D} G_{\mu\nu}\sigma_\mu^{(q)}\sigma_\nu^{(p)} \right\} \\ &= -N\overline{S}^2 \left[\frac{1}{2}\left(\frac{h}{4} - \frac{g}{2}\right) - \frac{h\beta\overline{S}^2}{8} \right] + \frac{\overline{S}^2}{4} H_{\rm spin} \equiv V_{\rm equalized}(X_1,\ldots,X_{DN}) , \end{aligned} \tag{23}$$

where $V_{\text{equalized}}$ is the function V when $\delta_q = 0$, for all q, and H_{spin} is as in Eq. (10). The dynamics then drives the system towards the minimum of $V = V_{\text{equalized}}$ [see Eq. (16)], which is, the minimum of a function that is indeed the hyperspin Hamiltonian (apart from a constant shift and a rescaling by the positive quantity $\overline{S}^2/4$, which is unavoidably present in unconstrained optimization and its value depends on the system parameters). The mapping between the hyperspin machine dynamics and the hyperspin Hamiltonian is rigorous only when the amplitudes are equalized. When the amplitudes are not perfectly equalized, there are deviations from this mapping, and the larger $\{\delta_q\}$ the more significant these deviations are. When some $\{\delta_q\}$ are nonzero but small, Eq. (23) from Eq. (22) is in general rewritten as

$$V(X_1, \ldots, X_{DN}) = -N\overline{S}^2 \left[\frac{1}{2} \left(\frac{h}{4} - \frac{g}{2} \right) - \frac{h\beta\overline{S}^2}{8} \right] + \frac{\overline{S}^2}{4} H_{\text{spin}} + v(X_1, \ldots, X_{DN}) \ , \tag{24}$$

where the correction $v = V - V_{\text{equalized}}$ includes all those terms where at least one power of δ_q appears. So now the minima of V are modified from those of $V_{\text{equalized}}$, and thus from those of the hyperspin Hamiltonian. But for small $\{\delta_q\}$, where V is close to $V_{\text{equalized}}$ up to corrections that are of the order of $\max_q\{|\delta_q|\}$, the minimum of V is expected to be close to that of $V_{\text{equalized}}$, and therefore H_{spin}.

This is precisely the scenario that we find numerically: For those cases where the ground state is not found at threshold, the less heterogeneous the amplitudes, the closer the retrieved energy is to the analytically determined minimum of H_{spin}.

B. Quantification of amplitude heterogeneity

The analysis in Eq. (24) suggests that the amplitude heterogeneity can be quantified by the *amplitude heterogeneity degree*, defined as ($\delta_q = S_q/\overline{S} - 1$)

$$A_{\text{het}} := \max_q\{\delta_q\} - \min_q\{\delta_q\} = \frac{\max_q\{S_q\} - \min_q\{S_q\}}{\overline{S}} \ , \tag{25}$$

where the smaller A_{het} the more homogeneous the amplitudes are. We can relate the behaviour of A_{het} as a function of h to that of the relative energy deviation $\Delta E/|E_{\text{GS}}|$, and show that the decrease of amplitude heterogeneity is intimately connected to the ability of the hyperspin machine to find to a good approximation the minimum of the spin Hamiltonian (see Supplementary Note 3).

Supplementary Note 3. Additional data on the energy minimization

To support our statements in the main text, we here provide additional data on the D-vector spin Hamiltonian minimization, i.e., Eq. (10) with $\mathbf{G} = \mathbb{1}_D$. We show in Supplementary Figs. 2-5 the relative PO energy deviation from the computed ground-state value $\Delta E/|E_{\text{GS}}|$ (panels with blue data) as a function of the pump amplitude deviation from threshold, where $\Delta E = E_{\text{PO}} - E_{\text{GS}}$. The value of E_{PO} is computed as the minimal spin energy retrieved from the steady-state PO amplitudes out of 50 repetitions of the PO network dynamics, for fixed simulation parameters. This is done to avoid detecting energy values of excited states, which in general may happen if the PO dynamics converges to local minima of the energy landscape. Notice that the reduction of amplitude heterogeneity ensures only that the mapping between the PO network cost function and the target cost function (coupled spins) is proper, but in general it does not ensure the convergence to the ground-state solution, since local minima of the spin Hamiltonian may always be encountered [4]. In our numerics, we checked that the retrieved spin configuration was indeed a minimum value (no value lower than the reported values of E_{PO} was ever detected). The value of E_{GS} is computed by minimizing the D-vector spin Hamiltonian for the same adjacency matrix by using `NMinimize` in Wolfram Mathematica. We choose three different graphs for different number of spins N and for $D = 2, 3, 4$, which are the XY, Heisenberg, and QCD model, respectively. In panels **a,d,g**, the adjacency matrix $\mathbf{J}$ represents a complete random K graph with $N = 10$, while in panels **b,e,h** it is a K graph with $N = 16$. In Supplementary Fig. 2 for the XY model, the insets show the XY phases $\{\varphi_q\}$ from the POs (blue dots) compared to the numerically computed phases (red circles) from the numerical minimization of the XY Hamiltonian with respect to the PO real quadratures. For the K graphs, the PO energy, by increasing the pump amplitude from the threshold h_{th}, starts from the value dictated by the eigenvector of the coupling matrix with maximal real part that deviates by approximately 10% from the numerically determined ground-state energy. As the pump amplitude increases, as in the main text, the energy rapidly approaches the computed ground state value, with a deviation below 0.05% in all cases, but it

does not exactly matches the numerically computed global minimum. Panels **c,f,i** represents instead a Möbius ladder graph [5] with antiferromagnetic interaction, which is a cubic circulant graph obtained by the adjacency matrix $\mathbf{J}$ with only nonzero elements $J_{qp} = J < 0$ for $|q-p| = 1$ and $|q-p| = N/2$. As evident, the energy from the PO phases exactly equals the computed ground-state energy for all scanned values of pump amplitude, both at threshold and well above it. This is an indication that the PO system finds exactly the global minimum of the selected optimization problem and, similarly to discrete (Ising) spin simulators for $D = 1$ [6, 7], it allows to conclude that the selected optimization problem belongs to the P class of computational complexity. The decrease of $\Delta E/|E_{\rm GS}|$ for increasing pump amplitude is compared to the behaviour of the heterogeneity degree ($A_{\rm het}$, panels with red data) in the same range of pump amplitude. For the K graphs, as evident, the fact that the energy from the hyperspin machine approaches the analytically determined minimum ($\Delta E/|E_{\rm GS}| \to 0$) is intimately related to the decrease of amplitude heterogeneity, which is is agreement with Eq. (24). For the Möbius ladder graph with $D \geq 2$, the heterogeneity degree is identically zero at any pump amplitude, and indeed the hyperspin machine finds exactly the ground-state energy of the corresponding spin Hamiltonian ($\Delta E/|E_{\rm GS}| = 0$).

It is important to remark that, while the reduction of amplitude heterogeneity steers the dynamical evolution of the POs towards the minimum of the desired spin Hamiltonian [see Eq. (24)] because it makes the mapping between the PO network cost function and the target cost function proper, the *presence* of amplitude heterogeneity in the steady state does not necessarily imply that the PO network does not find the correct ground state of the spin model. There are indeed special cases where the PO amplitudes in the steady state are heterogeneous, but the retrieved spin phases minimize the simulated spin Hamiltonian. Examples are easy (P) cases when the adjacency matrix represents a graph with boundaries (i.e., not all nodes have the same degree). A prototype example is the one dimensional ferromagnetic Ising ($D = 1$) chain with open boundaries, where the PO network prefers to store energy close to the central node (the POs close to the central node have the largest amplitude), minimizing the energy stored at the boundaries (the POs at the boundaries have the smallest amplitudes). Thus, the PO amplitudes are manifestly heterogeneous, but their phases yield the correct Ising solution (all amplitudes are positive, or all are negative). Another example is the antiferromagnetic 3D solid graph in Fig. 5**a,e,c,g** of the main text (see Supplementary Fig. 5), which also has boundaries, and indeed amplitudes are heterogenous but the hyperspin machine finds the correct ground state. For Ising models ($D = 1$) this can also happen when the ground-state Ising configuration presents domain walls (the Möbius ladder or antiferromagnetic nearest-neighbor graphs for some values of N are some examples). Also in these cases, increasing the pump amplitude decreases heterogeneity.

REFERENCES

[1] J. K. Kevorkian and J. D. Cole, *Multiple Scale and Singular Perturbation Methods* (Springer, New York, 1996).

[2] M. Calvanese Strinati, L. Bello, A. Pe'er, and E. G. Dalla Torre, "Theory of coupled parametric oscillators beyond coupled Ising spins," Phys. Rev. A **100**, 023835 (2019).

[3] J. Roychowdhury, "A global Lyapunov function for the coherent Ising machine," NOLTA, IEICE **13**, 227–232 (2022).

[4] T. Leleu, Y. Yamamoto, P. L. McMahon, and K. Aihara, "Destabilization of local minima in analog spin systems by correction of amplitude heterogeneity," Phys. Rev. Lett. **122**, 040607 (2019).

[5] R. K. Guy and F. Harary, "On the Möbius ladders," Canad. Math. Bull. **10**, 493–496 (1967).

[6] M. Calvanese Strinati, L. Bello, E. G. Dalla Torre, and A. Pe'er, "Can nonlinear parametric oscillators solve random Ising models?" Phys. Rev. Lett. **126**, 143901 (2021).

[7] K. P. Kalinin and N. G. Berloff, "Computational complexity continuum within Ising formulation of NP problems," Comm. Phys. **5**, 20 (2022).
