# Supplementary material for: Multidimensional hyperspin machine
Source: Nat Commun. 2022 Nov 25;13:7248. doi: 10.1038/s41467-022-34847-9 (PMC9700766; doi:10.1038/s41467-022-34847-9)
Supplement: Supplementary file 2 — Description of Additional Supplementary Files [file 41467_2022_34847_MOESM2_ESM.pdf]

File Name: Supplementary Movie 1
Description: Movie of the hyperspin dynamics for the random complete K graph with binary interaction strength. The hyperspin is represented in standard hyperspherical coordinates. In this case (XY spin), the arrows represent the hyperspin in polar coordinates retrieved from the PO amplitudes during the time evolution.

File Name: Supplementary Movie 2
Description: Movie of the hyperspin dynamics for the nearest-neighbour antiferromagnetic graph in a three-dimensional solid topology. The hyperspin is represented in standard hyperspherical coordinates. In this case (Heisenberg spin), the arrows represent the hyperspin in spherical coordinates retrieved from the PO amplitudes during the time evolution.

File Name: Supplementary Movie 3
Description: Movie of the hyperspin dynamics for the nearest-neighbour graph with random binary interaction in a three-dimensional solid topology. The hyperspin is represented in standard hyperspherical coordinates. In this case (QCD spin), the arrows and color of the outer spheres represent the vector in spherical coordinates and the scalar component, respectively, retrieved from the PO amplitudes during the time evolution.

File Name: Supplementary Movie 4
Description: Movie of the hyperspin dynamics during the dimensional annealing for the random complete K graph with binary interaction strength. The hyperspins initially tend to minimize the XY Hamiltonian for the selected graph on the xy-plane, and when the annealing starts, the spins gradually polarize along the x-axis, converging to a low-energy configuration of the Ising Hamiltonian for the same graph.
